# Heterogeneous Structure of Ni–Mo Nanoalloys Decorated on MoO_x_ for an Efficient Hydrogen Evolution Reaction Using Hydrogen Spillover

**DOI:** 10.1002/advs.202403752

**Published:** 2024-08-19

**Authors:** DongHoon Song, Jeonghan Roh, Jungwoo Choi, Hyein Lee, Gyungmo Koh, YongKeun Kwon, HyoWon Kim, Hyuck Mo Lee, MinJoong Kim, EunAe Cho

**Affiliations:** ^1^ Department of Materials Science and Engineering Korea Advanced Institute of Science and Technology (KAIST) 291 Daehak‐ro, Yuseong‐gu Daejeon 34141 Republic of Korea; ^2^ Hydrogen Research Department Korea Institute of Energy Research (KIER) 152 Gajeong‐ro, Yuseong‐gu Daejeon 34129 Republic of Korea; ^3^ Energy Engineering University of Science and Technology (UST) 217 Gajeong‐ro, Yuseong‐gu Daejeon 34113 Republic of Korea

**Keywords:** electrodeposition, heterogeneous Ni–Mo electrocatalyst, hydrogen evolution reaction, hydrogen molybdenum bronze, hydrogen spillover

## Abstract

Herein, a heterogeneous structure of Ni–Mo catalyst comprising Ni_4_Mo nanoalloys decorated on a MoO_x_ matrix via electrodeposition is introduced. This catalyst exhibits remarkable hydrogen evolution reaction (HER) activity across a range of pH conditions. The heterogeneous Ni–Mo catalyst showed low overpotentials only of 24 and 86, 21 and 60, and 37 and 168 mV to produce a current density of 10 and 100 mA cm^−2^ (η_10_ and η_100_) in alkaline, acidic, and neutral media, respectively, which represents one of the most active catalysts for the HER. The enhanced activity is attributed to the hydrogen spillover effect, where hydrogen atoms migrate between the Ni_4_Mo alloys and the MoO_x_ matrix, forming hydrogen molybdenum bronze as additional active sites. Additionally, the Ni_4_Mo facilitated the water dissociation process, which helps the Volmer step in the alkaline/neutral HER. Through electrochemical analysis, in situ Raman spectroscopy, and density functional theory calculations, the fast HER mechanism is elucidated.

## Graphical abstract

Ni–Mo electrocatalyst with a tailored nanostructure is synthesized by simple electrodeposition. The heterogeneous structure composed of Ni_4_Mo nanoalloys decorated on a MoO_x_ matrix displays efficient HER across a wide range of pH conditions: alkaline (1 m KOH), acidic (0.5 m H_2_SO_4_), and neutral (1 m PBS) electrolytes.

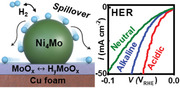

## 1 Introduction

Green hydrogen (H_2_) production by electrochemical water splitting has widely drawn attention as a promising technology for achieving carbon neutralization.^[^ ^1^, ^2^, ^3^, ^4^ ^]^ Hydrogen can efficiently serve as an energy carrier for the industrial and transportation sectors by converting surplus renewable power into clean hydrogen, compensating for the unstable and unpredictable nature of renewables.^[^ ^5^, ^6^, ^7^, ^8^, ^9^ ^]^ In particular, designing highly active electrocatalysts for the hydrogen evolution reaction (HER) is necessary to supply economical green hydrogen in water electrolysis systems. The catalytic materials for H_2_ production typically include platinum‐group metals (Pt, Ir, Rh, Ru, etc.) because of their outstanding adsorption and recombination properties of the active hydrogen species at the catalyst surface.^[^ ^10^, ^11^, ^12^, ^13^ ^]^ Due to the scarcity and high‐cost issue of Pt‐group metals,^[^ ^14^, ^15^, ^16^ ^]^ developing nonprecious‐group‐metals (NPGMs)‐based electrocatalysts of earth‐abundant materials is essential.^[^ ^17^, ^18^, ^19^, ^20^ ^]^ Among them, nickel–molybdenum (Ni–Mo) catalysts have attracted much attention due to their decent HER properties, attributed to the synergism between the Ni and Mo of the hypo‐hyper‐*d*‐electronic interactions.^[^ ^21^, ^22^, ^23^, ^24^, ^25^, ^26^, ^27^, ^28^ ^]^ Additional ternary elements were introduced to enhance the HER activity of Ni–Mo catalysts by further tuning the *d* orbital structure of Ni as well.^[^ ^29^, ^30^, ^31^, ^32^, ^33^ ^]^

Meanwhile, it is noteworthy that the heterogeneous structure of metal and metal (hydro)oxide catalysts could lead to a more efficient HER than metal alone.^[^ ^34^, ^35^, ^36^, ^37^ ^]^ The heterostructures have great potential to facilitate the HER kinetics; a representative example is the hydrogen spillover effect. The hydrogen spillover phenomenon refers to the migration of adsorbed hydrogen atoms in hetero‐catalysts from the metal surface to the other (hydro)oxide substrate or vice versa, which has been known to be effective for improving the HER activity.^[^ ^38^, ^39^, ^40^, ^41^, ^42^ ^]^ For instance, Park et al. reported synergistic effects from hydrogen spillover between Pt and tungsten suboxide, which enhanced the HER kinetics.^[^ ^40^ ^]^ Li et al. also suggested a fundamental viewpoint on the hydrogen spillover phenomenon between Pt alloys and CoP support, in which the HER activity could be significantly improved in the hybrid catalysts.^[^ ^43^ ^]^ In this regard, the Ni–Mo‐based catalysts have great potential to utilize the hydrogen spillover between heterogeneous structures because the hydrogen can effectively combine with the molybdenum oxides (MoO_x_), which could form a hydrogen molybdenum bronze phase.^[^ ^44^, ^45^, ^46^, ^47^ ^]^ Moreover, the hydrogen spillover effects could be helpful for efficient HERs in both acidic and alkaline media.^[^ ^26^, ^48^ ^]^

Nonetheless, constructing the desired nanointerfaces inside the microstructures for the Ni–Mo catalysts has still been complex and challenging.^[^ ^49^, ^50^, ^51^, ^52^ ^]^ In other words, suffering from synthetic complexity, the Ni–Mo catalysts have only been explored in the composition of binary metals, forming Ni–Mo alloys, and simply tuning the electronic structures of the Ni.^[^ ^53^, ^54^, ^55^, ^56^, ^57^ ^]^ Only a few reports have focused on the hydrogen spillover effects in heterogeneous Ni–Mo structures.^[^ ^26^, ^48^ ^]^ The role of the heterogeneous structures in Ni–Mo catalysts is also still being determined.

Herein, we report a heterogeneous structure of a Ni–Mo catalyst favorable for hydrogen spillover composed of Ni_4_Mo nanoalloys decorated on the MoO_x_ matrix by a simple electrodeposition process. By finely adjusting the deposition conditions, we examined the importance of the heterostructure in the Ni–Mo catalysts on the HER activity. The heterogeneous structure of the Ni–Mo exhibited an excellent HER activity in 1 m potassium hydroxide (KOH), 0.5 m sulfuric acid (H_2_SO_4_), and 1 m phosphate buffer solution (PBS). Electrochemical analysis, in situ Raman spectroscopy, and density functional theory (DFT) calculations demonstrate that the faster HER kinetics would be attributed to the hydrogen spillover from the Ni_4_Mo nanoalloys to the MoO_x_ matrix, which results in the formation of hydrogen molybdenum bronze (H_x_MoO_3_) serving as additional active sites for the HER. We provide a facile method to synthesize the hetero‐structure of the Ni–Mo catalyst and shed light on the mechanisms for the efficient HER in the nano‐scale interfaces.

## 2 Results and Discussion

### 2.1 Characterization of the Ni–Mo Electrodes

**Figure 1:**
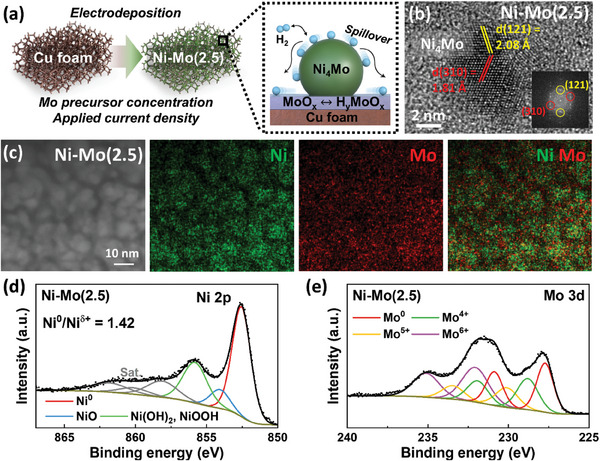
a) Schematic of the Ni–Mo(2.5) and the heterogeneous microstructure of the Ni–Mo electrocatalysts (inset). b) HRTEM image of the Ni–Mo nanoalloy with SAED pattern (inset) in Ni–Mo(2.5). c) Cross‐sectional microstructures observed by FIB‐TEM and the corresponding EDS mapping images of Ni–Mo(2.5). d) XPS Ni 2p and e) Mo 3d narrow scan for the electrodeposited catalyst in the Ni–Mo(2.5).

**Figure** **1**a shows a brief schematic of the electrodeposition process for the Ni–Mo electrodes. To obtain the desired Ni–Mo heterostructure, Ni–Mo catalysts with different Ni/Mo ratios were synthesized by electrodeposition on pristine Cu foam at 0.5 A cm^−2^ (Table S1, Supporting Information). All surface shapes of the prepared electrodes did not differ significantly depending on the Mo precursor concentration and applied current density, showing a similar morphology to the pristine Cu foam (Figure S1, Supporting Information). The thickness of the uniform Ni–Mo catalyst layer was estimated to be 233 ± 10 nm with a catalyst loading of 0.4 mg cm^−2^ on the Cu substrate (Figure S2, Supporting Information). In addition, X‐ray diffraction (XRD) patterns for all electrodes matched well with the crystal structure of the Cu substrate, irrespective of the electrodeposition conditions (Figure S3, Supporting Information), suggesting that the Ni–Mo‐based electrodes are composed predominantly of amorphous microstructures.

The Mo content in the electrodeposited Ni–Mo layers did not change significantly as the Mo precursor concentration increased, but the oxygen (O) content increased, and the Ni decreased (Figure S4, Supporting Information). It is widely known that Mo could be deposited either as metallic Mo, Ni–Mo alloys (e.g., Ni_4_Mo), or molybdenum oxides (MoO_x_) as suggested in the electrodeposition mechanism^[^ ^58^, ^59^, ^60^, ^61^ ^]^ (Equation 1):

(1) $$\begin{array}{ccc} & & \mathrm{pMo}{O_{4}}^{2-}+\mathrm{qHCi}t^{3-}+rH^{+}\\ & & \;\to \;{\left[{\left(\mathrm{Mo}{O_{4}}^{2-}\right)}_{p}{\left(\mathrm{HCi}t^{3-}\right)}_{q}{\left(H^{+}\right)}_{r}\right]}^{\left(2p+3q-r\right)-} \end{array}$$

(2) $$\mathrm{NiCi}t^{-}+2\;e^{-}\to \mathrm{Ni}+\mathrm{Ci}t^{3-}$$

(3) $$\begin{array}{ccc} & & {\left[\left(\mathrm{Mo}O_{4}\right)\mathrm{HCitH}\right]}^{4-}+4Ni^{2+}+3H_{2}O+14e^{-}\\ & & \;\to \;\mathrm{Mo}+4\mathrm{Ni}\left(Ni_{4}\mathrm{Mo}\right)+7OH^{-}+\mathrm{HCi}t^{3-} \end{array}$$

(4) $$\begin{array}{c} {\left[\left(\mathrm{Mo}O_{4}\right)\mathrm{HCitH}\right]}^{4-}+H_{2}O+2e^{-}\to \mathrm{Mo}O_{2}+3OH^{-}+\mathrm{HCi}t^{3-} \end{array}$$

(5) $$\begin{array}{c} \mathrm{Mo}{O_{4}}^{2-}+\mathrm{NiCi}t^{-}+H_{2}O+2e^{-}\to \mathrm{Mo}O_{3}+\mathrm{Ni}+2OH^{-}+\mathrm{Ci}t^{3-} \end{array}$$

Based on this, the increase of the O content is related to the formation of molybdenum oxide (MoO_x_) rather than the metallic Mo or Ni–Mo alloys. Thus, increasing the Mo precursor concentration could bring the dominant MoO_x_‐based phase in the electrodeposited Ni–Mo catalysts. These results imply that the microstructure may vary depending on the Ni/Mo ratio, which would be attributable to differences in the proportions and distributions of the Ni and Mo metallic and oxide phases.

To detect the expected Ni–Mo alloy phase, meanwhile, the XRD pattern for a specific Ni–Mo electrode was plotted in log‐scale intensity (Figure S5, Supporting Information). However, the main peaks for the Ni_4_Mo overlapped with the strong intensity from the Cu substrate, which makes it challenging to identify the phase by XRD. Nonetheless, the broad characteristic arouses together ≈43.4°, 50.5°, and 74.2° which stand for (111), (200), (220) of the Cu foam, might be ascribed from the amorphous‐dominant microstructures for the electrodeposited Ni–Mo catalysts onto Cu substrate.

In this regard, the cross‐sectional microstructures with *d*‐spacing of the crystallite planes were carefully observed to clarify the phase in the Ni–Mo catalysts by FIB‐TEM. Among the Ni–Mo electrodes, only Ni–Mo(2.5) exhibited clear interfaces between the nanoalloys and matrix as desired (Figure 1b). This was indicated by the planes of Ni_4_Mo (310) and Ni_4_Mo (121) with a *d*‐spacing of 1.81 and 2.08 Å, respectively, confirmed by the SAED pattern (inset). FIB‐TEM EDS mapping images, reflecting the elemental distribution of the Ni and Mo, support the presence of distinctive interfacial microstructures in the heterogeneous Ni–Mo‐based nanoalloys decorated on a Mo‐based matrix (Figure 1c). However, the Ni–Mo(X) catalysts electrodeposited from the higher Mo precursor concentration (X = 5, 10, 20) were hardly distinguishable for the interface (Figure S6, Supporting Information) and showed relatively homogeneous Ni and Mo distributions compared to the Ni–Mo(2.5) sample (Figure S7, Supporting Information). The Ni/Mo ratio affected the microstructure, and the applied current density also had a significant role. When a low current density of 0.1 A cm⁻^2^ was applied at the same Mo precursor concentration (Ni–Mo(2.5)‐L), a homogeneous distribution of the Ni and Mo was observed without distinct interface formation (Figures S6 and S7, Supporting Information). In the Ni–Mo(2.5)‐H, electrodeposited at a higher current density of 1 A cm⁻^2^, discernible nano‐crystallites were observed in the HRTEM images. However, these nano‐crystallites were also composed of pure Ni and Mo, exhibiting a d‐spacing of 2.04 and 1.58 Å for the Ni (111) and Mo (200) planes, respectively, in addition to Ni_4_Mo.^[^ ^50^, ^62^ ^]^ Thus, it is inferred that the specific conditions of the Mo precursor concentration and the applied current density could lead to the desired heterogeneous microstructures for electrodeposited Ni–Mo catalysts.

The chemical bonding states of the surface Ni in the Ni–Mo electrodes were investigated by X‐ray photoelectron spectroscopy (XPS). The Ni 2p XPS narrow spectrum has a major peak at 852.6 eV assigned to metallic Ni (Ni^0^), along with two oxidized Ni peaks (854.1 and 855.8 eV corresponding to NiO and either Ni(OH)_2_ or NiOOH, respectively) and satellite peaks (Figure 1d).^[^ ^18^ ^]^ Because the metallic Ni peak would be derived from the Ni‐based nanoparticles, the areal ratio of the metallic‐to‐oxidized Ni (Ni^0^/Ni^δ+^) could specify the Ni's heterogeneity in each sample. It is noteworthy that the Ni–Mo(2.5) had the highest metallic‐to‐oxidized Ni ratio (Ni^0^/Ni^δ+^ = 1.42), which might be attributed to the configuration of the Ni‐based nanoparticles (Figure 1d; Figure S8, Supporting Information). These quantitative Ni XPS spectra confirmed that the specific electrodeposition conditions could induce a rational structure for the heterogeneous Ni–Mo catalysts. In addition, the Mo 3d XPS narrow spectrum in all the Ni–Mo electrodes was deconvoluted into four major peaks at 227.7, 228.9, 230.1, and 232.1 eV corresponding to Mo^0^, Mo^4+^, Mo^5+^, and Mo^6+^, respectively (Figure 1e).^[^ ^50^ ^]^ The Mo 3d XPS scans also imply the presence of metallic and oxidized Mo species, which correspond to either Mo or Mo‐Ni alloys and MoO_x_, respectively (Figure S9, Supporting Information). All these results of the morphological microstructures by TEM images and chemical bonding states by XPS corroborated that the heterogeneous structure of Ni_4_Mo nanoalloys decorated on MoO_x_ could be successfully obtained in the Ni–Mo(2.5) catalyst.

### 2.2 Electrochemical Properties for the Hydrogen Evolution Reaction

**Figure 2:**
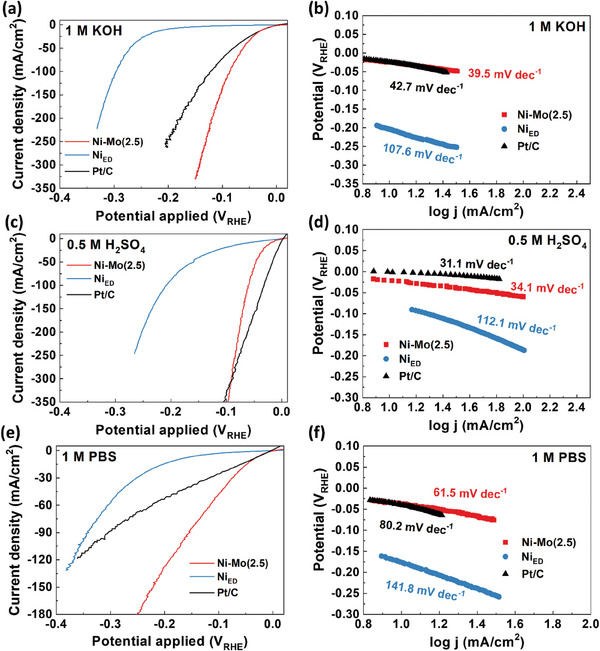
HER polarization and Tafel plots for Ni–Mo(2.5), Ni_ED_, and Pt/C catalysts in wide‐pH electrolytes; 0.1 m KOH a,b), 0.5 m H_2_SO_4_, c,d), and 1 m PBS e,f), respectively. Each LSV curve was measured with a scan rate of 2 mV s^−1^ (iR‐corrected).

HER polarization curves of the heterogeneous Ni–Mo(2.5) were measured in comparison to those of electrodeposited Ni (Ni_ED_) and commercial Pt/C (**Figure** **2**) across a range of pH conditions (1 m KOH, 0.5 m H_2_SO_4_, and 1 m PBS). Overpotentials required to produce a current density of 10 and 100 mA cm^−2^ are denoted as η_10_ and η_100,_ respectively. Notably, the Ni–Mo(2.5) exhibited excellent HER activity in the alkaline media showing overpotentials only of 24 (η_10_) and 86 mV (η_100_) which are superior to those of the commercial Pt/C (η_10_ = 24 mV and η_100_ = 122 mV) (Figure 2a). Furthermore, the Ni–Mo(2.5) showed remarkable HER properties in acidic (Figure 2c) and neutral electrolytes (Figure 2e). In the acidic HER, Ni–Mo(2.5) showed overpotentials only of 21 (η_10_) and 60 mV (η_100_), comparable to those of the commercial Pt/C (η_10_ = 3 mV and η_100_ = 33 mV) in the high current density region. In the neutral HER, Ni–Mo(2.5) had an outstanding HER activity with overpotentials of 37 (η_10_) and 168 mV (η_100_) which are lower than those of the Pt/C (η_10_ = 41 mV and η_100_ = 329 mV). The overpotentials are listed in Table S2 (Supporting Information).

The improvement of the HER activity can be associated with an increase in the active surface area or an enhancement of the intrinsic activity. Because the Ni–Mo(2.5)‐L had the lowest metallic‐to‐oxidized Ni ratio (Ni^0^/Ni^δ+^ = 0.58) among the given electrodeposition conditions (Figure S8, Supporting Information), Ni–Mo(2.5)‐L was selected as a control group for the homogeneous Ni–Mo structures. The double‐layer capacitance (C_dl_) of all Ni–Mo catalysts was measured using the cyclic voltammetry (CV) technique (Figure S10, Supporting Information). The calculated C_dl_ values were similar (≈14 mF cm^−2^) for all Ni–Mo catalysts and Ni_ED_ (Figure S11 and Table S3, Supporting Information). Thus, the improved HER activity of the Ni–Mo(2.5) would originate from the intrinsic property of the catalyst rather than from the electrochemical surface area.

Tafel plots were collected (Figure 2b,d,f) to investigate the intrinsic activity of the prepared catalysts. It is widely accepted that the HER occurs on a catalyst surface through a multi‐step process (Equation 6),^[^ ^34^, ^63^ ^]^ where “M” refers to the metals, and “M‐H” refers to the hydrogen‐adsorbed metals. In contrast to the acidic HER where the H adsorption takes place from a proton‐rich environment (Equations 6 and 7), the alkaline/neutral HER should involve the water dissociation step to supply the reactants which results in a sluggish activity (Equations 7 and 9).^[^ ^35^, ^36^, ^37^ ^]^ Moreover, it is regarded as the rate‐determining step (RDS).

(6) $$\mathrm{Volmer}\;\mathrm{reaction}\left(\mathrm{in}\;\mathrm{acid}\right)\;:H^{+}+e^{-}+M\to M-H$$

(7) $$\left(\mathrm{in}\;\mathrm{alkaline}/\mathrm{neutral}\right)\;:H_{2}O+e^{-}+M\to M-H+OH^{-}$$

(8) $$\mathrm{Heyrovsky}\;\mathrm{reaction}\left(\mathrm{in}\;\mathrm{acid}\right)\;:M-H+H^{+}+e^{-}\to H_{2}+M$$

(9) $$\left(\mathrm{in}\;\mathrm{alkaline}/\mathrm{neutral}\right)\;:M-H+H_{2}O+e^{-}\to H_{2}+OH^{-}+M$$

(10) $$\mathrm{Tafel}\;\mathrm{reaction}\left(\mathrm{in}\;\mathrm{acid}\right)\;:M-H+M-H\to H_{2}+2M$$

The HER mechanism on the catalyst can be roughly speculated from the experimental Tafel slope; ≈120, 40, or 30 mV dec^−1^ indicates that the rate of the HER is determined by the Volmer, Heyrovsky, or Tafel reaction, respectively.^[^ ^63^, ^64^, ^65^ ^]^ The Ni–Mo(2.5) had the smallest Tafel slope of 39.5 and 61.5 mV dec^−1^ in 1 m KOH and 1 m PBS, respectively, indicating faster HER kinetics compared to the pure Ni_ED_ (107.6 and 141.8 mV dec^−1^, respectively) and Pt/C (43.8, and 80.2 mV dec^−1^, respectively). In 0.5 m H_2_SO_4_, the Ni–Mo(2.5) showed an almost comparable Tafel slope (34.1 mV dec^−1^) to the Pt/C (31.1 mV dec^−1^), which is regarded as the fastest acidic HER kinetics among the electrocatalysts. Compared to the values of each Tafel slope for the pristine Ni_ED_ in the alkaline/neutral media (107.6/141.8 mV dec^−1^), those of the Ni–Mo(2.5) (39.5/61.5 mV dec^−1^) were remarkably decreased (Figure 2b,f). Introducing Mo could effectively accelerate the water dissociation process, which was validated in other Ni, Mo‐based electrocatalysts.^[^ ^66^, ^67^ ^]^ Based on the overall Tafel slopes of Ni–Mo(2.5), it could be speculated that the HER pathway follows the Volmer–Tafel reaction in the acidic media and the Volmer–Heyrovsky reaction in the alkaline/neutral electrolyte.^[^ ^65^ ^]^

The HER polarization curves of the other Ni–Mo batches were compared in Figure S12 (Supporting Information). The heterogeneous Ni–Mo structure in Ni–Mo(2.5) had the highest HER activity among the other Ni–Mo electrodes. The long‐term durability of Ni–Mo(2.5) was estimated by chronopotentiometry at a constant current density of −100 mA cm^−2^ for 20 h in each electrolyte (Figure S13 and Table S4, Supporting Information). In comparison to Ni_ED_ and Pt/C, the Ni–Mo(2.5) showed a negligible overpotential increase (82–82 mV) in contrast to the Ni_ED_ (304–323 mV) and Pt/C (126–251 mV). In 0.5 M H_2_SO_4_, all prepared catalysts exhibited decent stability without activity loss. In 1 m PBS, the Ni–Mo(2.5) had a smaller overpotential increase (163–171 mV) than that of Ni_ED_ (360–445 mV) and Pt/C (313–341 mV), demonstrating excellent HER stability across a range of pH conditions for the heterogeneous Ni–Mo catalysts as well. The surface morphology of Ni–Mo(2.5) after the durability test observed by SEM and cross‐sectional images were well maintained (Figure S14, Supporting Information). Also, post‐XPS analysis for the Ni–Mo(2.5) after the chronopotentiometry test shows that most of the catalyst surface was oxidized (Figure S15, Supporting Information). However, the surface charges of Ni and Mo after the Ar etching were almost identical to those of the as‐prepared sample (Figure 1d,e). Given that measuring the ex situ XPS after HER could instantly lead to the oxidized surface from the inevitable air exposure, the post‐XPS analysis confirms again the excellent HER stability for Ni–Mo(2.5).

The Ni–Mo(2.5) also showed excellent cell performance, exhibiting only an overpotential of 370 mV to reach 100 mA cm^−2^ in the alkaline water electrolysis, which presents great practical applicability (Figure S16, Supporting Information). To the best of our knowledge, among the previously reported Ni–Mo‐based electrocatalysts for HER, the Ni–Mo(2.5) has the smallest overpotentials across a range of pH conditions (Table S5, Supporting Information). All these electrochemical results demonstrated that the heterogeneous microstructures of the Ni_4_Mo nanoalloys decorated on the MoO_x_ matrix have an outstanding HER activity.

### 2.3 Hydrogen Spillover on the NiMo nanoalloys decorated on the MoO_x_ Catalyst

**Figure 3:**
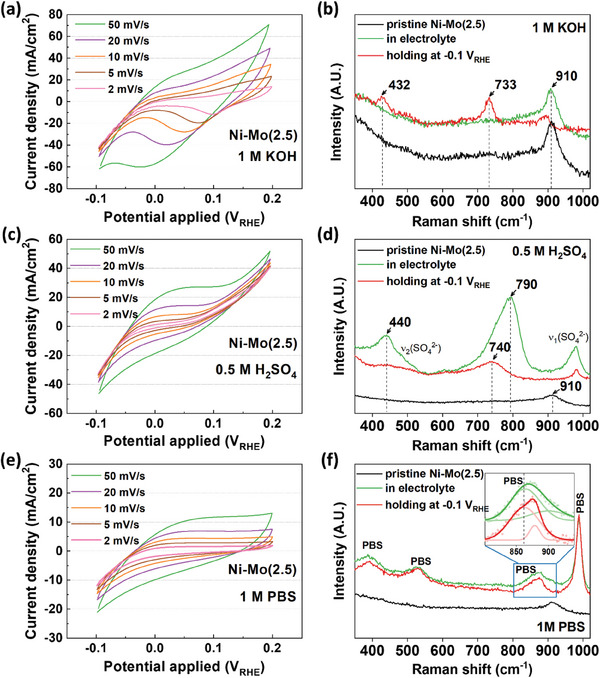
Cyclic voltammetry from −0.1 to 0.2 V_RHE_ and in situ Raman spectroscopy for Ni–Mo(2.5) in wide‐pH electrolytes; a,b) 0.1 m KOH, c,d) 0.5 m H_2_SO_4_, and e,f) 1 m PBS, respectively.

To elucidate the morphological effects related to the specific configuration of the Ni–Mo nanoalloys decorated on the MoO_x_ matrix, cyclic voltammetry (CV) was conducted in alkaline, acidic, and neutral environments (**Figure** **3**). The CV was measured from −0.1 to 0.2 V_RHE_ at various scan rates from 2 to 50 mV s^−1^. Interestingly, the Ni–Mo(2.5) showed pseudocapacitive behaviors in all electrolytes when increasing the scan rate (Figure 3a,c,e). In contrast, the behavior was not observed in the Ni–Mo(2.5)‐L and pure Ni_ED_ (Figure S17, Supporting Information). It is well known that the pseudocapacitive behavior would be attributed to the reversible redox process of MoO_x_/H_y_MoO_x_ which results from hydrogen insertion/extraction, respectively.^[^ ^68^, ^69^ ^]^ In other words, the MoO_x_ matrix in the Ni–Mo(2.5) is capable of forming hydrogen molybdenum bronze (H_y_MoO_x_) by the insertion of hydrogen from the electrolytes as follows (Equation 11):

(11) $$\mathrm{Mo}O_{x}+yH^{+}+ye^{-}↔H_{y}\mathrm{Mo}O_{x}$$

To confirm the formation of the H_y_MoO_x_ in the Ni–Mo(2.5), in situ Raman spectroscopy was performed (Figure 3b,d,f). The Ni–Mo(2.5) was immersed in each electrolyte for 10 min, and then the potential from −0.15 to 0.15 V_RHE_ was applied for 40 cycles at a scan rate of 40 mV s^−1^ for surface stabilization. During the measurement, the Ni–Mo(2.5) was exposed under a constant potential of −0.1 V_RHE_ in each electrolyte. In all the electrolytes, the as‐prepared Ni–Mo(2.5) showed a broad peak at 910 cm^−1^, which is assigned to a vibration of the terminal Mo═O stretching mode.^[^ ^70^, ^71^ ^]^ In the 0.5 m H_2_SO_4,_ where the hydrogen adsorption could be observed due to the high proton concentration (Figure 3d), the peaks at 790 and 440 cm^−1^ can be assigned to the Mo_2_‐O and Mo_3_‐O stretching in the H_x_MoO_3_.^[^ ^72^, ^73^, ^74^, ^75^, ^76^ ^]^ When a HER potential of −0.1 V_RHE_ was applied to the electrode, the peak at 790 cm^−1^ was left shifted, which is closely associated with a weakening of the force constant of the bond, indicating that the structure of the H_x_MoO_3_ is somewhat distorted with the increased inter‐layer spacing as a result of hydrogen intercalation during HER.^[^ ^73^, ^76^, ^77^ ^]^ In 1 M KOH, the spectra changed little when immersed, but a pronounced peak was observed at 733 cm^−1^ associated with the H_x_MoO_3_ phase (Figure 3b). Although there was no apparent change for the spectra in 1 m PBS compared to the pristine, the negative peak shift of MoO_x_ was also confirmed during the HER (Figure 3f). All these results of the pseudocapacitive behavior and in situ Raman spectra consistently corroborate the formation of hydrogen molybdenum bronze (H_y_MoO_x_) at the MoO_x_ matrix in the heterogeneous structure of Ni–Mo(2.5).

### 2.4 DFT Calculations

**Figure 4:**
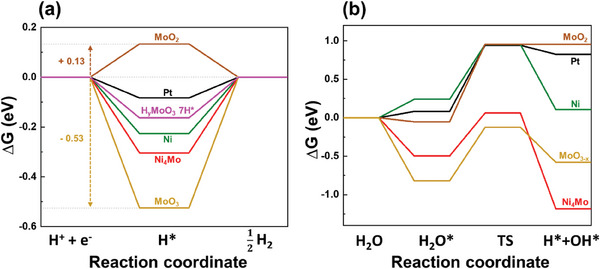
a) Gibbs free energy diagram for the H adsorption/desorption reaction on the modeled systems of Pt, Ni, MoO_2_, MoO_3_, H_x_MoO_3_‐7H*, and Ni_4_Mo. Each calculation energy is listed in Table S6 (Supporting Information). b) Gibbs free energy diagram for H_2_O adsorption/dissociation reaction on the modeled systems of Pt, Ni, MoO_2_, MoO_3‐x_, and Ni_4_Mo. Each energy value is listed in Table S7 (Supporting Information).

DFT calculations were conducted to mechanistically clarify the efficient HER of Ni–Mo(2.5) across a range of pH conditions. The details of each modeling structure of Pt, Ni, Ni_4_Mo, MoO_2_, and MoO_3_ are depicted in the supplementary information (Figure S18, Supporting Information). Based on the modeled structures, the H adsorption energy (ΔG_H*_) and water dissociation barrier were calculated (**Figure** **4**). Regarding the H adsorption energy (ΔG_H*_), it is widely known that the material for which the ΔG_H*_ approaches 0 has the ideal adsorption energy for an efficient HER.^[^ ^12^, ^78^ ^]^ Notably, the H adsorption energy (ΔG_H*_) of MoO_2_ and MoO_3_ was 0.13 and −0.53 eV (Figure 4a), respectively, which indicates that the MoO_2_ would not adsorb protons. At the same time, MoO_3_ has strong H adsorption properties, possibly resulting in the hydrogen molybdenum bronze phase (H_y_MoO_3_). Based on the modeling, the formation energy of the hydrogen molybdenum bronze (H_y_MoO_3_‐nH^*^, *n* = 1–8) was calculated to verify the thermodynamic stability of the phase (Figure S19, Supporting Information). The surface Pourbaix energy diagram was calculated to verify the thermodynamic stability of the phase (Figure S20, Supporting Information). The theoretical surface Pourbaix diagram, derived from DFT‐based electronic structure calculations, represents the thermodynamic relative stability of the system. Our calculations build on previous work by the Nørskov group.^[^ ^79^ ^]^ As shown in Figure S20 (Supporting Information), the analysis confirms that the state with seven hydrogen atoms adsorbed on the modeled MoO_3_ (H_y_MoO_3_‐7H*) is thermodynamically stable between −0.4 and 0 V_RHE_. In contrast, H_y_MoO_3_‐8H becomes stable at potentials below −0.4 V_RHE_. The experimental environment indicates that the reaction potential remains above −0.4 V_RHE_, thereby verifying the reliability of our modeled H_y_MoO_3_‐7H configuration.

To compare the theoretical activity of the HER, we investigated the H adsorption energy (ΔG_H*_) of the H_y_MoO_3_‐7H* and Ni_4_Mo, including Pt and Ni as references (Figure 4a). The hydrogen adsorption energies of Pt and Ni were −0.08 and −0.23 eV, respectively, consistent with the findings of a previous study.^[^ ^78^ ^]^ The adsorption energy of H_y_MoO_3_‐7H* was −0.16 eV which is located between that of the Pt and Ni (Table S6, Supporting Information). The Ni_4_Mo had a stronger H adsorption compared to the Ni. Thus, the H adsorption results are consistent with the experimental HER tendency in the acidic media, where hydrogen adsorption/desorption mainly determines the reaction activity. In addition, the diffusion barrier of hydrogen adsorbed on Ni_4_Mo was calculated, and it was very low, with an average of 0.13 eV, as shown in Figure S21 (Supporting Information). Meanwhile, H_2_O dissociation is regarded as an essential step that should precede the supply of reactants for the HER in the alkaline and neutral electrolytes.^[^ ^80^, ^81^ ^]^ A Gibbs free energy diagram illustrates the H_2_O adsorption/dissociation on MoO_2_, MoO_3‐x_, Pt, Ni, and Ni_4_Mo (Figure 4b). In the case of MoO_3_, the H_2_O adsorption/dissociation was analyzed at the oxygen vacant MoO_3_ (MoO_3‐x_) because the MoO_3_ has deficient catalytic sites for the calculation. As a result, the adsorption energy of H_2_O on Ni_4_Mo (−0.50 eV) is stronger than on Pt (0.08 eV), Ni (0.24 eV), and MoO_2_ (−0.05 eV). Compared to Pt (0.86 eV), Ni (0.71 eV), MoO_2_ (1.01 eV), and MoO_3‐x_ (0.69 eV), the activation energy of the H_2_O* dissociation was lower for Ni_4_Mo (0.56 eV), which indicates that the H_2_O dissociation reaction is facilitated in Ni_4_Mo (Table S7, Supporting Information).

### 2.5 Overall HER Mechanism

Based on the above calculations, it is noteworthy that the formation of H_y_MoO_x_ would be closely related to the hydrogen spillover from the NiMo nanoalloys following the suggested mechanism below (Equation 12).

(12) $$Ni_{4}\mathrm{Mo}+H^{+}\left(H_{2}O\right)+e^{-}\to Ni_{4}\mathrm{Mo}-H\left(+OH^{-}\right)$$

(13) $$\begin{array}{ccc} & & yNi_{4}\mathrm{Mo}-H+\mathrm{Mo}O_{x}\\ & & \;\to \;yNi_{4}\mathrm{Mo}+H_{y}\mathrm{Mo}O_{x}-nH^{*}\left(x=3,n=7\right) \end{array}$$

(14) $$H_{y}\mathrm{Mo}O_{x}-nH^{*}\left(x=3,n=7\right)\to y/2H_{2}+\mathrm{Mo}O_{x}$$

Protons from the electrolyte would be adsorbed at the Ni_4_Mo alloy particles followed by hydrogen spillover onto the adjacent MoO_x_ matrix, resulting in the formation of hydrogen molybdenum bronze (H_y_MoO_x_‐nH^*^ (x = 3, *n* = 7)). Due to the low diffusion barrier of hydrogen adsorbed on Ni_4_Mo, the recovery of a clean Ni_4_Mo surface would occur and serve as the re‐adsorption sites of the hydrogen. In acid, the bronze phase could also be formed from sufficient protons in the electrolyte and act as additional active sites instantaneously. In the alkaline and neutral media, water dissociation should precede the supply of protons, which makes the Volmer step determine the HER kinetics. This suggested spillover mechanism corresponds well to the Tafel slope results in Figure 2, explaining the Volmer–Heyrovsky and Volmer–Tafel reactions in alkaline/neutral and acidic electrolytes, respectively. That is to say, the hetero‐structures of the Ni_4_Mo nanoalloy decorated on the MoO_x_ matrix have synergistic effects for boosting the HER across a range of pH conditions.

**Figure 5:**
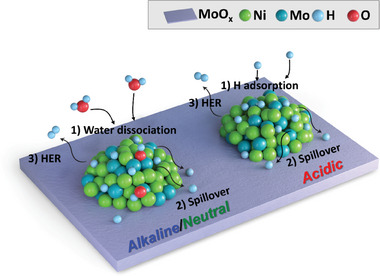
Schematic illustration of the suggested HER mechanisms on the heterogeneous Ni–Mo catalysts over the wide‐pH electrolytes.

Finally, we identified the origin of the HER activity for the heterogeneous Ni–Mo catalysts over a wide pH range. **Figure** **5** shows a schematic illustration of the suggested mechanism. Due to the low hydrogen diffusion barrier, the hydrogen molybdenum bronze phase (H_y_MoO_3_) would be easily formed due to either the strong hydrogen adsorption or the spillover from the Ni_4_Mo alloy. The hydrogen adsorption energy of the modeled stable bronze phase (H_y_MoO_3_‐7H*) was located between Pt and Ni. This result indicates that the activity of Ni–Mo(2.5) is higher than that of Ni–Mo(2.5)‐L and Ni_ED_ and worse than Pt in the Volmer‐Tafel reaction mechanism. In an alkaline/neutral environment, we confirmed that the H_2_O adsorption and dissociation are facilitated better in Ni_4_Mo compared to Pt, Ni, MoO_2_, and MoO_3‐x_. Again, it would be reasonable to suggest that a facile H_2_O dissociation occurs on Ni_4_Mo, leading to the fast diffusion of the generated H* from Ni_4_Mo to MoO_3_. This spillover of H* onto MoO_3_ results in the formation of hydrogen molybdenum bronze which contributes to the excellent HER activity. Furthermore, we confirmed theoretically that the H_2_O dissociation occurs more effectively on the Ni_4_Mo compared to the Pt. While its activity was inferior to that of the Pt in the acidic environment, we identified an enhanced activity compared to that of the Pt in the alkaline/neutral environments where H_2_O dissociation has a pivotal role. Overall, the experimental and theoretical analyses well matched the suggested mechanism.

## 3 Conclusion

In summary, we elucidated the microstructural effects of the binary Ni–Mo electrocatalysts for the HER. Heterogeneous Ni–Mo structures were successfully obtained by finely controlling the electrodeposition conditions, such as precursor concentration and applied current density. The Ni–Mo(2.5) composed of quasi‐crystalline Ni_4_Mo nanoparticles decorated on MoO_x_ matrix showed remarkable HER activity in alkaline, acid, and neutral media. The H insertion/extraction was observed by the pseudocapacitive behavior in the Ni–Mo(2.5) only. The in situ Raman spectroscopy confirmed the formation of the hydrogen molybdenum bronze (H_y_MoO_x_) phase during the HER, which was closely related to the excellent catalytic activity. The DFT calculations revealed that the bronze phase (H_y_MoO_x_) would be formed by the H adsorption from the protons in the acid and serve as additional active sites for the HER with a formation of the stable H_y_MoO_3_‐7H^*^ structure. The H_2_O dissociation process responsible for the proton supply in the alkaline/neutral media was facilitated in the Ni_4_Mo phase, accelerating the Volmer–Heyrovsky reaction pathway. The hydrogen spillover formed the bronze phase (H_y_MoO_x_) in the higher‐pH electrolytes from the Ni_4_Mo surface, which was confirmed by the strong H adsorption property of the catalyst surface and the low diffusion barrier of hydrogen. We suggest the overall HER mechanisms across a range of pH conditions for heterogeneous Ni–Mo catalysts composed of Ni_4_Mo nanoalloys decorated on MoO_x_. This study provides a rational design principle for binary electrocatalysts for highly efficient HER in universal‐pH electrolytes and sheds light on mechanisms for state‐of‐the‐art technology.

## 4 Experimental Section

### Chemicals and Materials

All chemicals were purchased and directly utilized without any further purification; nickel chloride hexahydrate (NiCl_2_∙6H_2_O, 98%), trisodium citrate dihydrate (C_6_H_5_Na_3_O_7_∙2H_2_O, 99%), and sodium molybdate dihydrate (Na_2_MoO_4_∙2H_2_O, 99%), potassium hydroxide (KOH, 85%), sulfuric acid (H_2_SO_4_, 95%), phosphate‐buffered saline (PBS, pH 6.3–6.7), sodium hydroxide (NaOH, 95%), and potassium chloride (KCl, 99.5%) were purchased from Junsei Chemicals. Deionized water with a specific resistance of 18.2 MΩ cm was obtained using a Milipore‐DQ5 purification system. The commercial Pt/C (20 wt.%) was purchased from Tanaka (TKK).

### Preparation of the Ni–Mo Electrodes by Electrodeposition

Ni–Mo electrodes were fabricated by electrodeposition using a two‐electrode cell consisting of a 1 cm^2^ copper (Cu) foam and a pure nickel plate as the cathode and anode, respectively. The naturally formed Cu oxide film and any contaminants on the Cu foam surface were removed by immersing in 3 m H_2_SO_4_ for 5 min.

With controlling the Mo precursor concentration of Na_2_MoO_4_∙2H_2_O from 1.25, 2.5, 5, 10, and 20 mm, the electrodeposition was conducted in a bath containing 0.1 m NiCl_2_∙6H_2_O and 0.2 m C_6_H_5_Na_3_O_7_∙2H_2_O. The applied current density was fixed to 0.5 A cm^−2^ for 5 min. The distance between the cathode and anode was 1 cm. The bath solution was stirred at 300 rpm and maintained at 30 °C. The electrodes obtained from each bath condition were denoted as Ni–Mo(1.25), Ni–Mo(2.5), Ni–Mo(5), Ni–Mo(10), and Ni–Mo(20), respectively, according to the Mo precursor concentration. For comparison, Ni–Mo(2.5)‐L and Ni–Mo(2.5)‐H (L and H denote low and high current density, respectively) were prepared with applied current densities of 0.1 and 1 A cm^−2^, respectively, while maintaining the same bath concentration as Ni–Mo(2.5). A Ni electrode was also prepared by electrodepositing Ni at Cu foam without the use of Mo precursor. The electrodeposition conditions are shown in Table S1 (Supporting Information).

### Catalyst Characterization

The morphologies of the electrodeposited catalysts were investigated by scanning electron microscopy (SEM) and transmission electron microscopy (TEM). The phase information was analyzed using X‐ray diffraction (XRD). The compositions of the entire catalyst and the catalyst surface were measured by an energy dispersive spectrometer (EDS) by SEM and the XPS measurement, respectively. The chemical state of the catalysts was examined by X‐ray photoelectron spectroscopy (XPS) measurement.

### Electrochemical Measurement

All tests were carried out using a three‐electrode system at room temperature. The electrodeposited catalysts on Cu foam were used as the working electrode. A graphite rod was used as the counter electrode, and a Hg/HgO and saturated calomel electrode were used as the reference electrodes for the alkaline and neutral/acidic electrolytes, respectively. The 1 m KOH, 1 m PBS, and 0.5 m H_2_SO_4_ solutions were purged with argon gas for stable electrolytes.

A Pt/C electrode was also prepared for comparison. Catalyst ink was prepared by mixing 10 mg of Pt/C, 900 uL of isopropyl alcohol (IPA), 900 uL of DI water, and 200 uL of Nafion solution (5 wt.%, Sigma Aldrich). Then, 100 uL of catalyst ink was spray‐coated onto a 1 cm^2^ Ni foam with a loading amount of 0.5 mg cm^−2^ (porosity; >99.5%, density; 350 g m^−2^, Sq‐300 Invisible Co.). The electrode was dried in a vacuum for 30 min at 60 °C. All potentials were calibrated with respect to the reversible hydrogen electrode (RHE).

For the hydrogen evolution reaction (HER) measurement, the polarization curves were obtained by scanning the potential from 0.05 to −0.5 V_RHE_ at a scan rate of 2 mV s^−1^. Chronopotentiometry was carried out at −100 mA cm^−2^ for 100 h to estimate the stability of the catalysts. All curves were reported with iR compensation according to the electrolyte resistance measured by electrochemical impedance spectroscopy (EIS). The EIS test was conducted in the frequency range of 10 kHz–0.1 Hz at −0.1 V_RHE_ (1 m KOH), −0.05 V_RHE_ (0.5 m H_2_SO_4_), and −0.15 V_RHE_ (1 m PBS).

### Alkaline Water Electrolysis Cell Measurement

The HER performance of the prepared Ni–Mo electrode was evaluated in an alkaline water electrolysis cell. The bare Ni foam was used as the anode for the oxygen evolution reaction (OER). Each area of the active electrodes was 1 cm^2^, and the electrolyte for the 5 m KOH solution was used at 60 °C. The polarization curves were measured from 1.2 to 2.55 V_RHE_ at a scan rate of 2 mV s^−1^.

### Computational Details

Density functional theory (DFT) calculations were performed using the Vienna Ab‐initio Software Package (VASP).^[^ ^82^ ^]^ A generalized gradient approximation (GGA) with the revised Perdew–Burke–Ernzerhof (RPBE) functional was used to describe the exchange‐correlation interactions. The projector‐augmented‐wave (PAW) method with a plane wave up to an energy of 480 eV was used to consider the interaction between the valence electrons and the ionic core.^[^ ^83^, ^84^ ^]^ The convergence criteria for the electronic structure and the geometry optimization were 10^−5^ eV and 0.03 eV Å^−1^, respectively. The spin polarization and the dipole correction along the z‐direction were applied.^[^ ^85^ ^]^ The Hubbard DFT+U method with an effective U_eff_ of 6.3 eV was applied to treat the Mo d‐orbitals for the MoO_3_ systems.^[^ ^86^, ^87^, ^88^ ^]^ The climbing‐image nudged elastic band method (CI‐NEB) was used to calculate the activation barrier.^[^ ^89^ ^]^ The p(2 × 2)‐Pt(111), p(2 × 2)‐Ni(111), p(1 × 1)‐Ni_4_Mo(121), p(1 × 2)‐MoO_2_(011), and p(2 × 2)‐MoO_3_(001) surfaces with >20 Å of vacuum were modeled as reactive surfaces to simulate the properties of these electrocatalysts. The bottom two layers of the slabs were fixed during the geometry optimization, and the other layers were relaxed.

## Conflict of Interest

The authors declare no conflict of interest.

## Supporting information

Supporting Information

## Data Availability

The data that support the findings of this study are available from the corresponding author upon reasonable request.
