# Supplementary material for: Heterogeneous Structure of Ni–Mo Nanoalloys Decorated on MoOx for an Efficient Hydrogen Evolution Reaction Using Hydrogen Spillover
Source: Adv Sci (Weinh). 2024 Aug 19;11(39):2403752. doi: 10.1002/advs.202403752 (PMC11497032; doi:10.1002/advs.202403752)
Supplement: Supplementary file 1 — Supporting Information [file ADVS-11-2403752-s001.docx]

**Supplementary Information**

**Heterogeneous structure of Ni-Mo nanoalloys decorated on MoOx for an efficient hydrogen evolution reaction using hydrogen spillover**

DongHoon Song^a+^, Jeonghan Roh^a+^, Jungwoo Choi^a^, Hyein Lee^a^, Gyungmo Koh^a^, YongKeun Kwon^a^, HyoWon Kim^a^, Hyuck Mo Lee^a^, MinJoong Kim^b^* and EunAe Cho^a^*

*^a^Department of Materials Science and Engineering, Korea Advanced Institute of Science and Technology (KAIST), 291 Daehak-ro, Daejeon 34141, Republic of Korea*

*^b^Hydrogen Research Department, Korea Institute of Energy Research (KIER), 152 Gajungro, Daejeon 34129, Yuseong-gu, Daejeon 34129, Republic of Korea*

+ equally contributed to this work.

*Corresponding author.

E-mail address: mj.kim@kier.re.kr (MinJoong Kim), eacho@kaist.ac.kr (EunAe Cho)


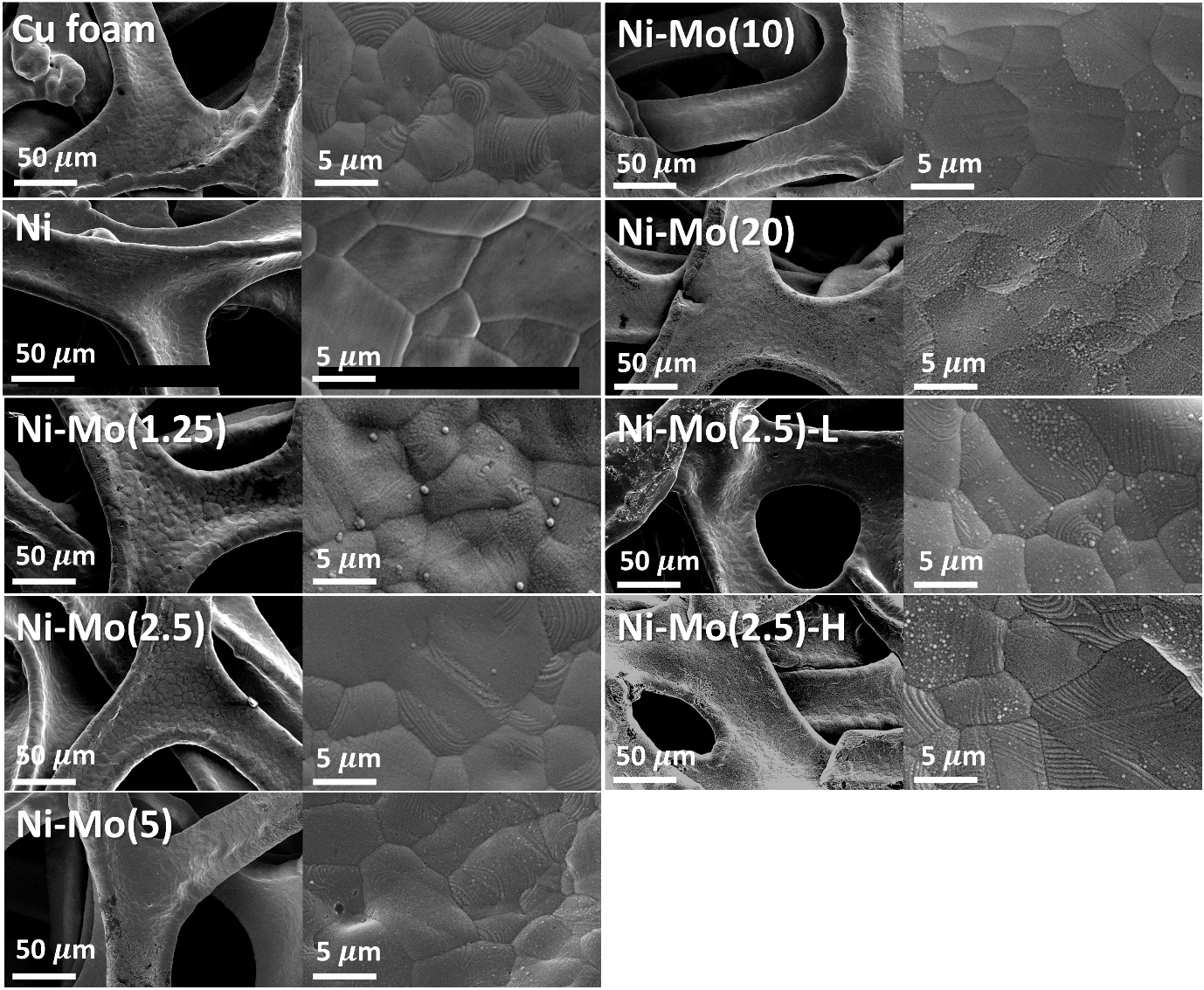


**Figure S1.** SEM images of the Cu foam, electrodeposited Ni, and Ni-Mo electrodes fabricated under various electrodeposition conditions.


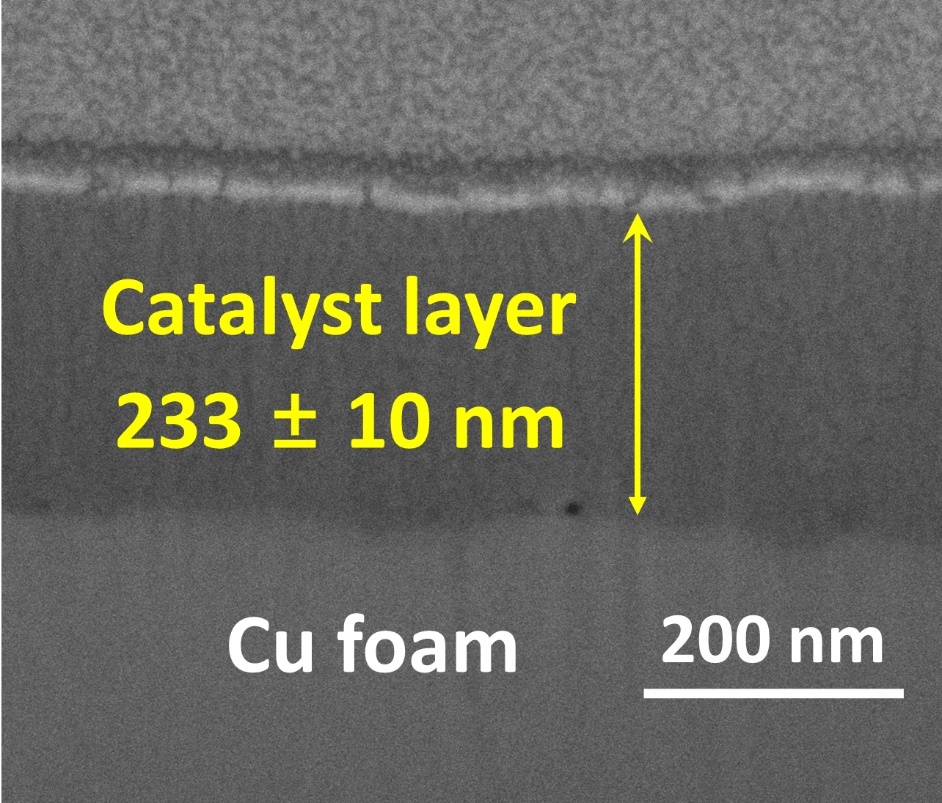


**Figure S2.** A cross-sectional SEM image of Ni-Mo(2.5) deposited on a Cu substrate. After the electrodeposition, the total loading of the catalyst layer was estimated to be 0.4 mg cm^-2^.

**Figure S3.** X-ray diffraction (XRD) patterns for the electrodeposited Ni-Mo electrodes. Regardless of the electrodeposition conditions, only Cu peaks were observed from the Cu substrate.

**
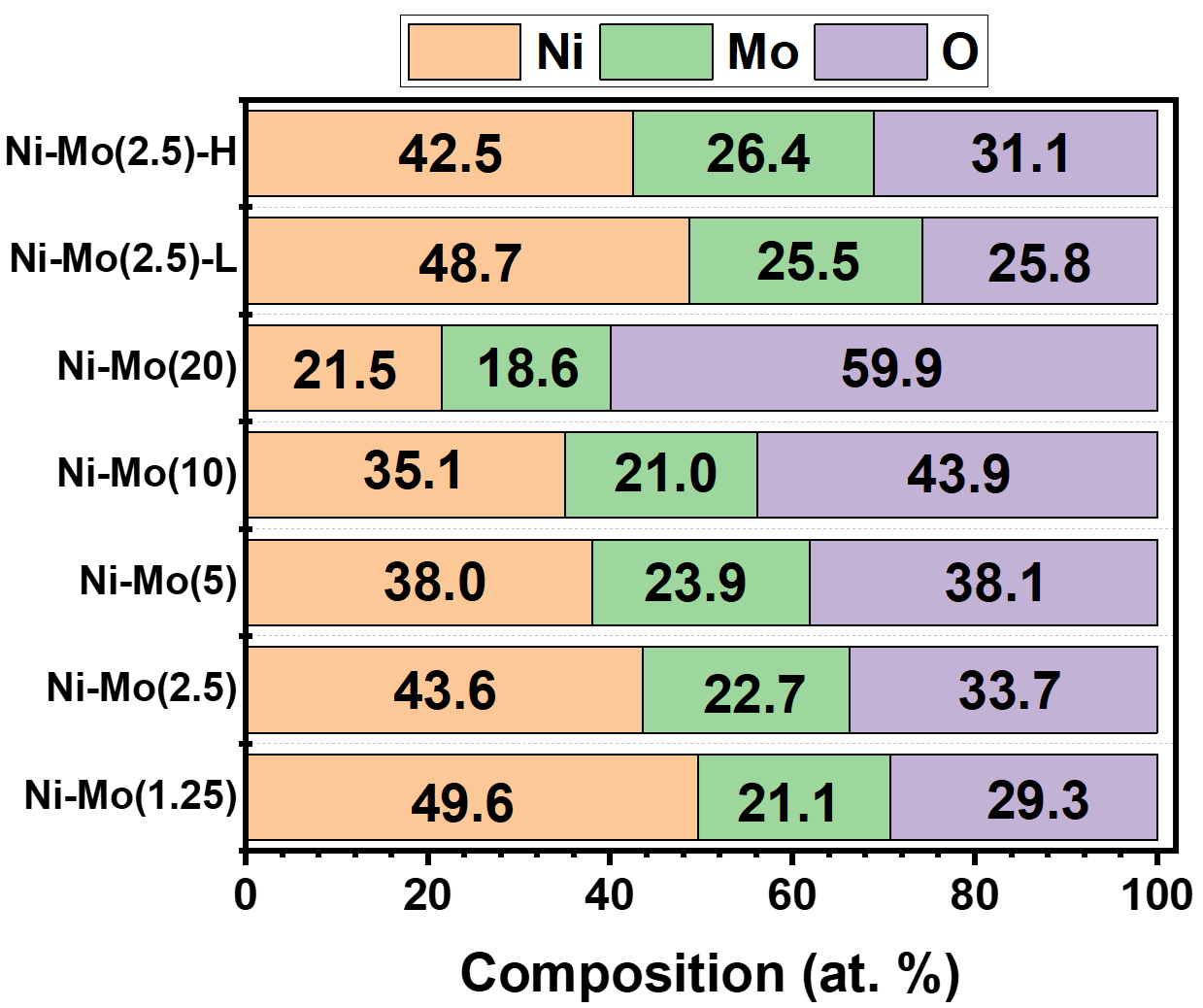
**

**Figure S4.** Elemental composition of the Ni, Mo, and O for the Ni-Mo electrodes from SEM energy dispersive spectroscopy (EDS) analysis. The atomic ratios of Ni, Mo, and O were collected from the entire electrode region where the signals were enough to be stable.

**Figure S5.** Log-scale plot of X-ray diffraction (XRD) patterns for the Ni-Mo(2.5).


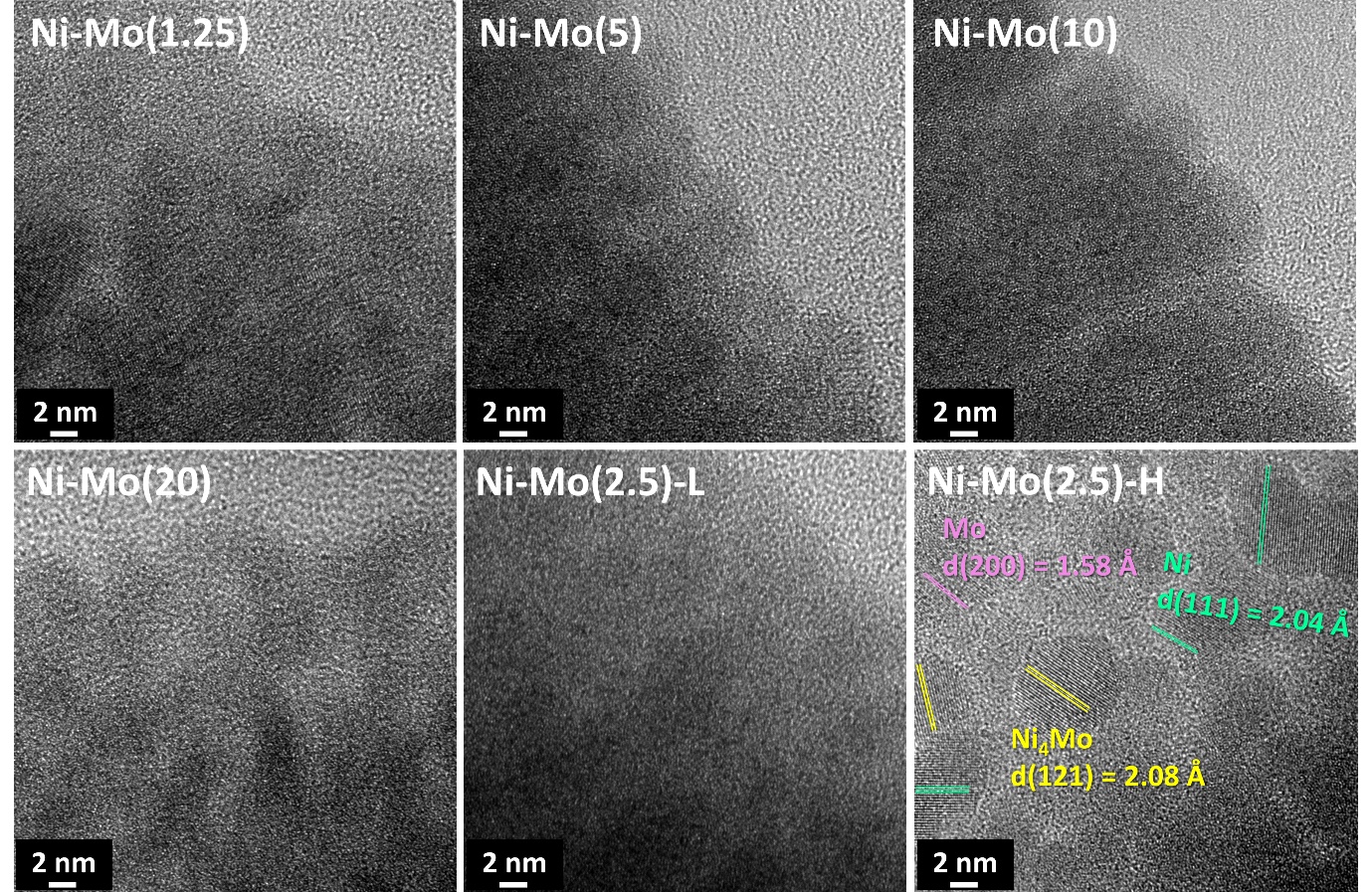


**Figure S6.** HRTEM images with d-spacings of the crystallites in the Ni-Mo electrodes.


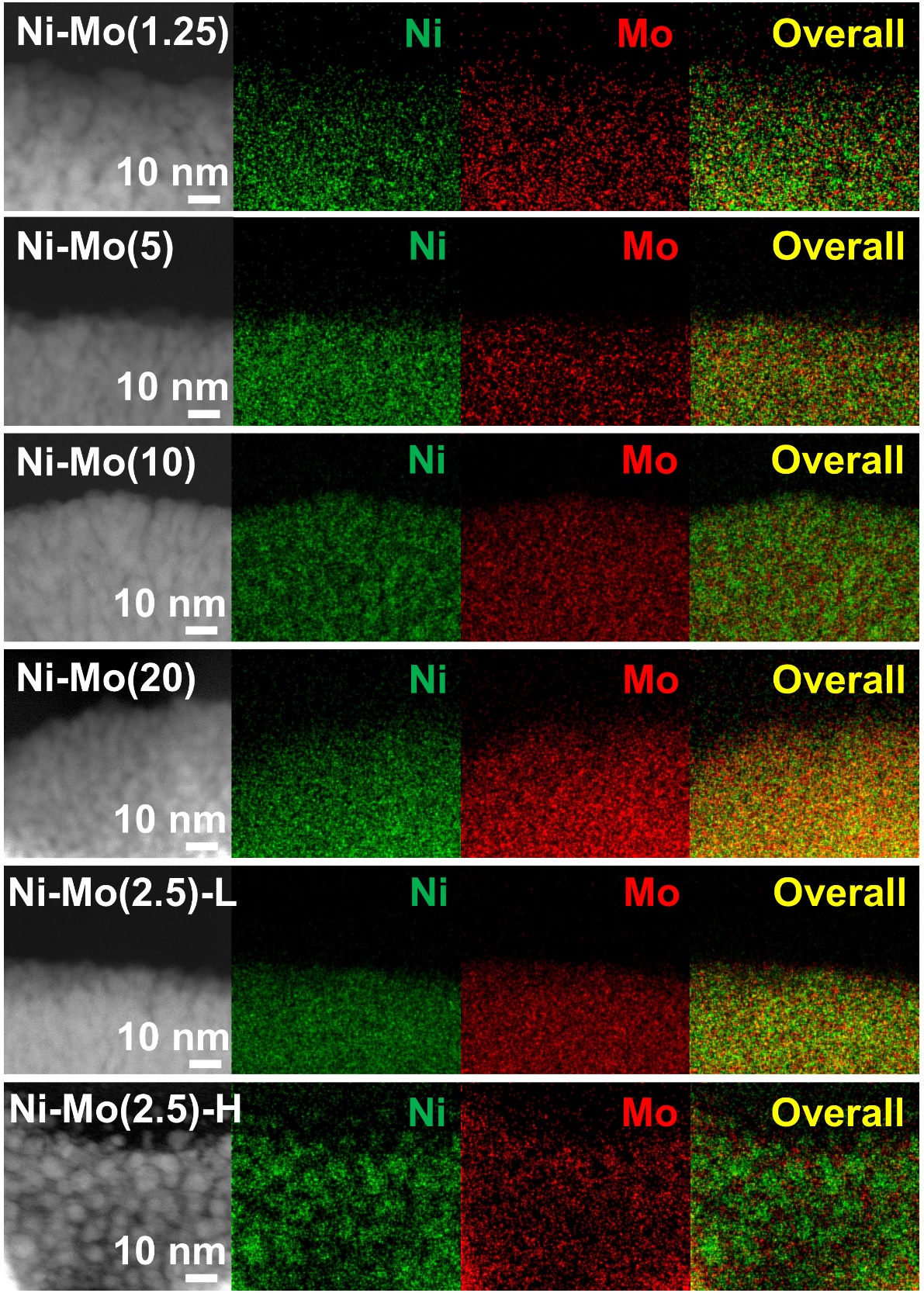


**Figure S7.** The cross-sectional microstructures observed by FIB-TEM EDS mapping images of the Ni-Mo electrodes.


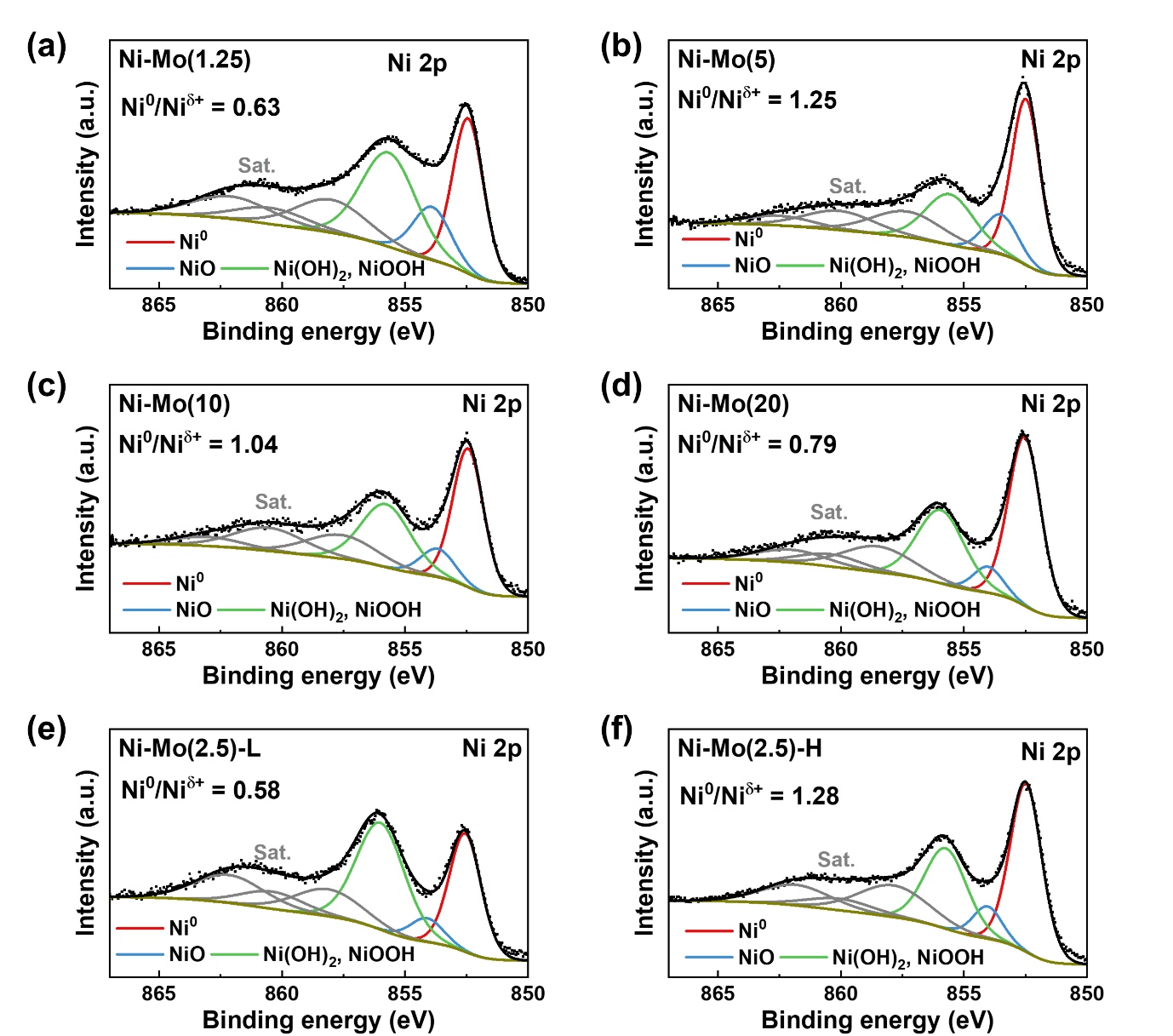


**Figure S8.** XPS Ni 2p narrow scans for the catalysts in other Ni-Mo electrodes; (a) Ni-Mo(1.25), (b) Ni-Mo(5), (c) Ni-Mo(10), (d) Ni-Mo(20), (e) Ni-Mo(2.5)-L, (f) Ni-Mo(2.5)-H.


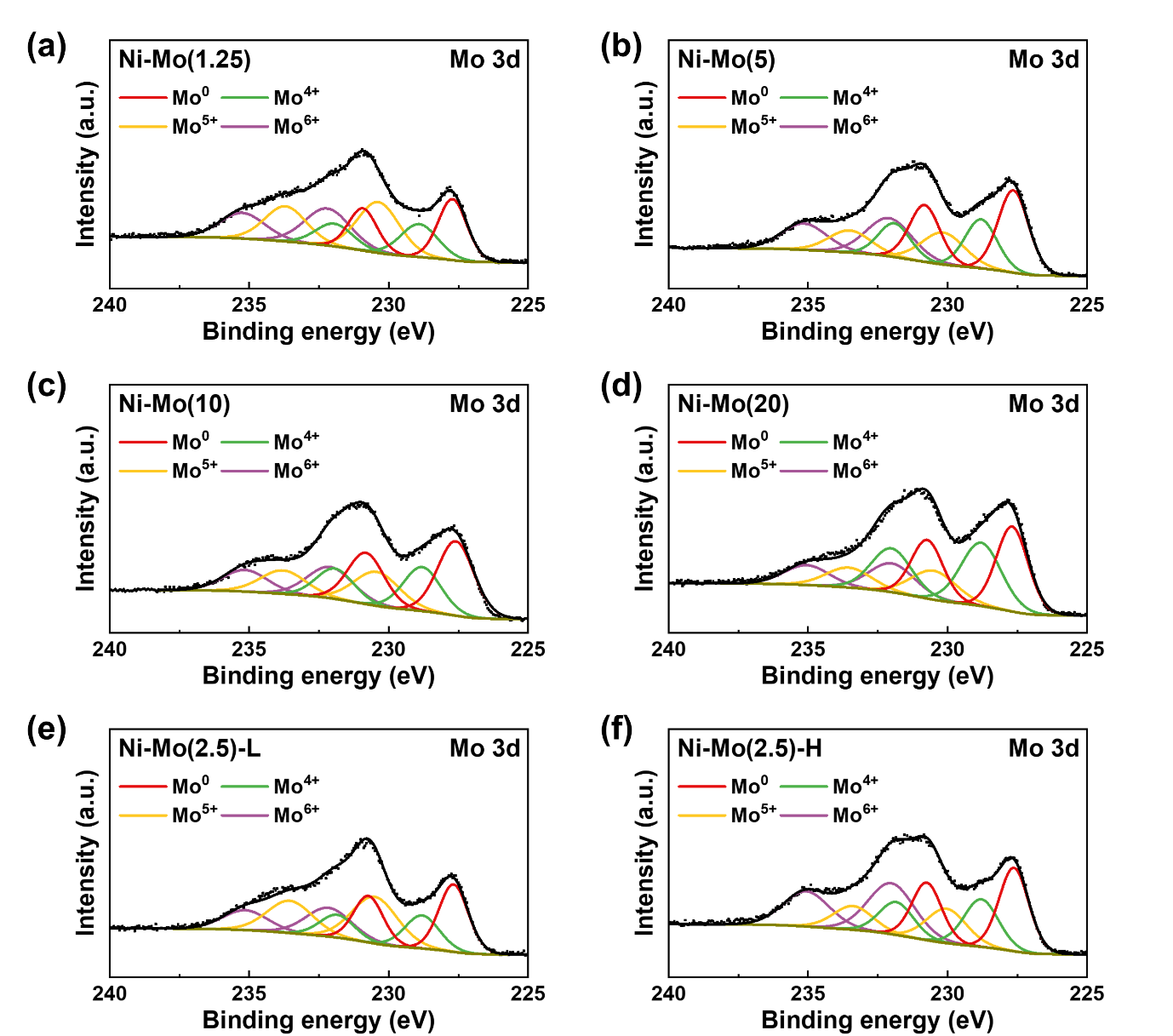


**Figure S9.** XPS Mo 3d narrow scans for the catalysts in other Ni-Mo electrodes; (a) Ni-Mo(1.25), (b) Ni-Mo(5), (c) Ni-Mo(10), (d) Ni-Mo(20), (e) Ni-Mo(2.5)-L, (f) Ni-Mo(2.5)-H.

**
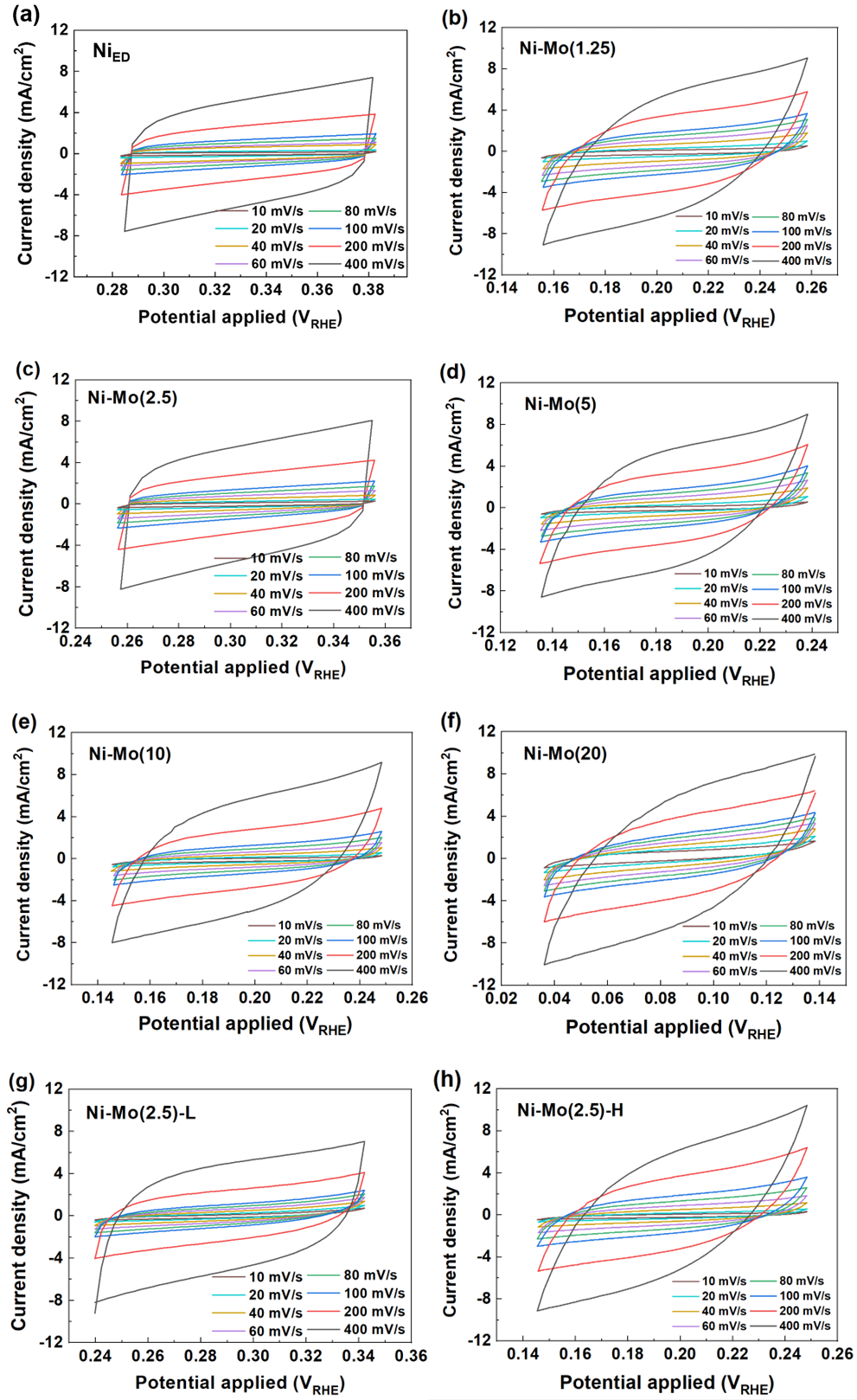
**

**Figure S10.** Cyclic voltammograms (CV) measured at scan rates from 10 to 400 mV/s for the (a) Ni_ED_, (b) Ni-Mo(1.25), (c) Ni-Mo(2.5), (d) Ni-Mo(5), (e) Ni-Mo(10), (f) Ni-Mo(20), (g) Ni-Mo(2.5)-L, and (h) Ni-Mo(2.5)-H measured in 1 M KOH.

**
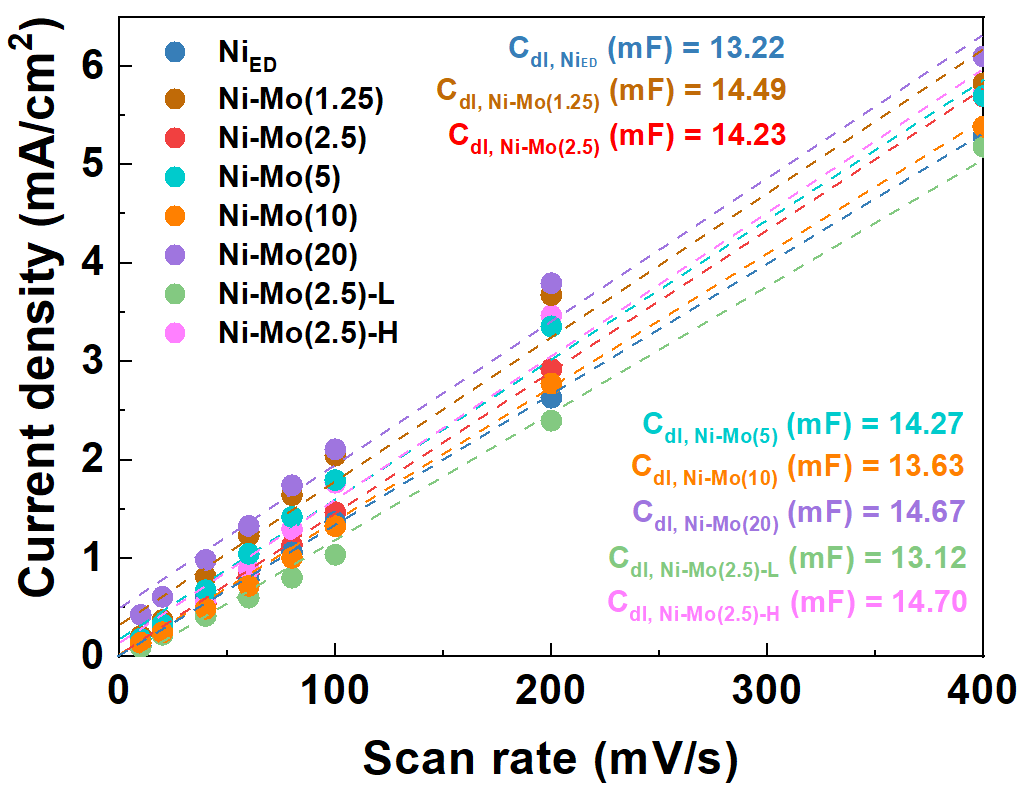
**

**Figure S11.** Capacitive current vs. scan rates plots for all Ni-Mo catalysts measured in 1 M KOH. The double-layer capacitance (C_dl_) values are listed in **Table S5**.

**
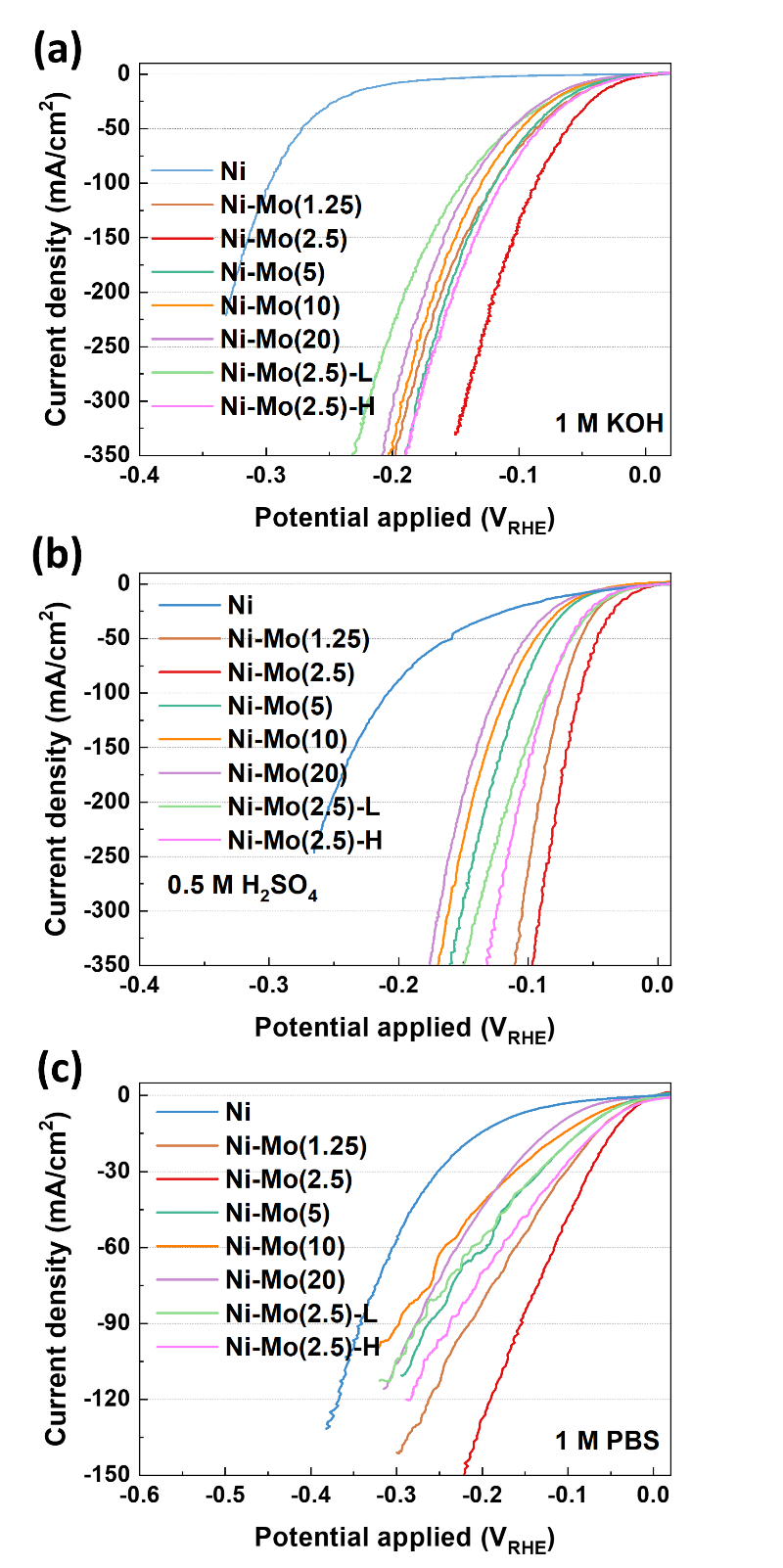
**

**Figure S12.** Electrochemical HER polarization curves of the as-prepared Ni-Mo electrodes. The LSV curves were measured in (a) 1 M KOH, (b) 0.5 M H_2_SO_4_, and (c) 1 M PBS.


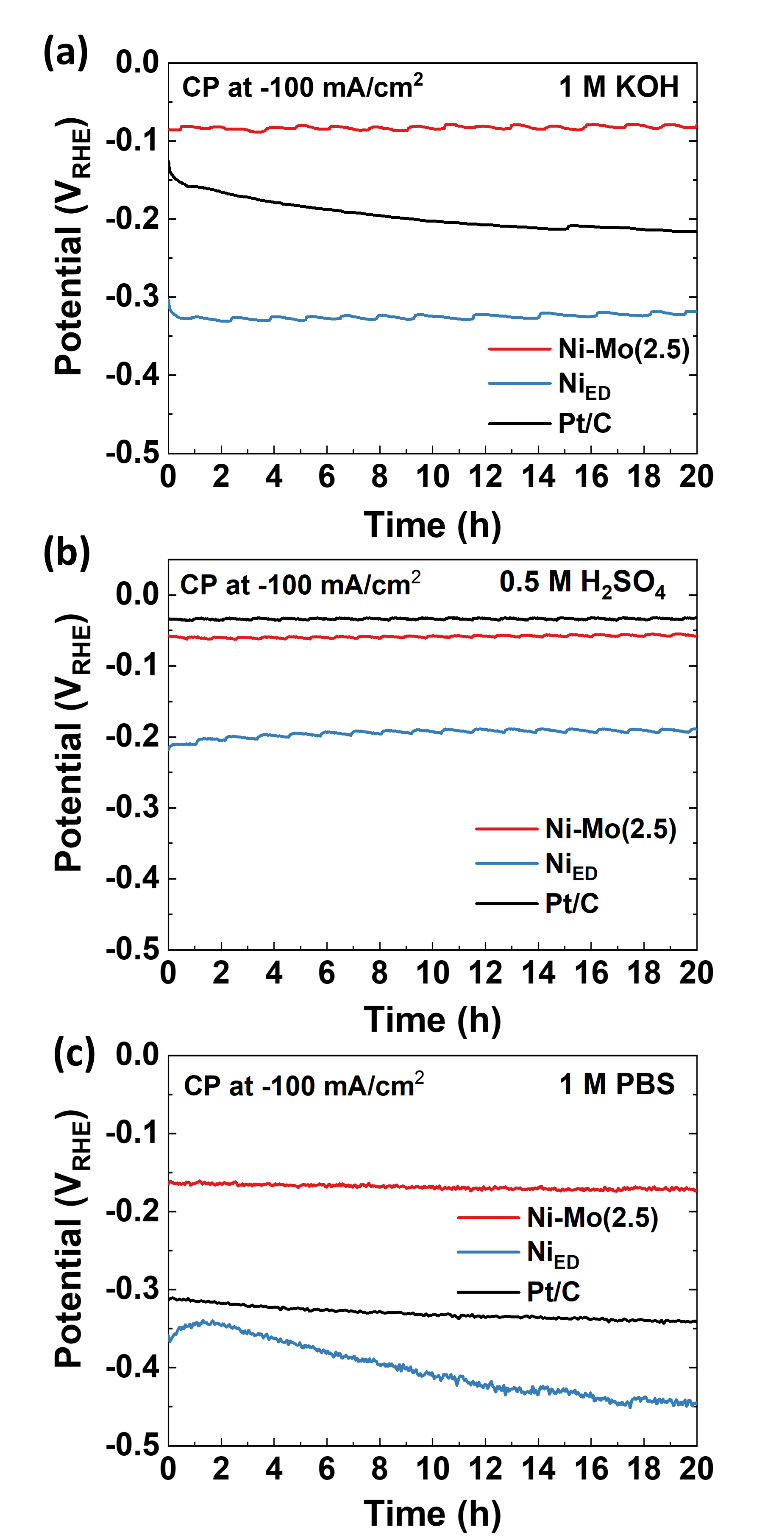


**Figure S13.** Chronopotentiometry curves for Ni-Mo(2.5), Ni_ED_, and Pt/C catalysts in wide-pH electrolytes: (a) 1 M KOH, (b) 0.5 M H_2_SO_4_, and (c) 1 M PBS. The durability test was conducted at a constant current density of -100 mA/cm^2^ for 20 h.

**
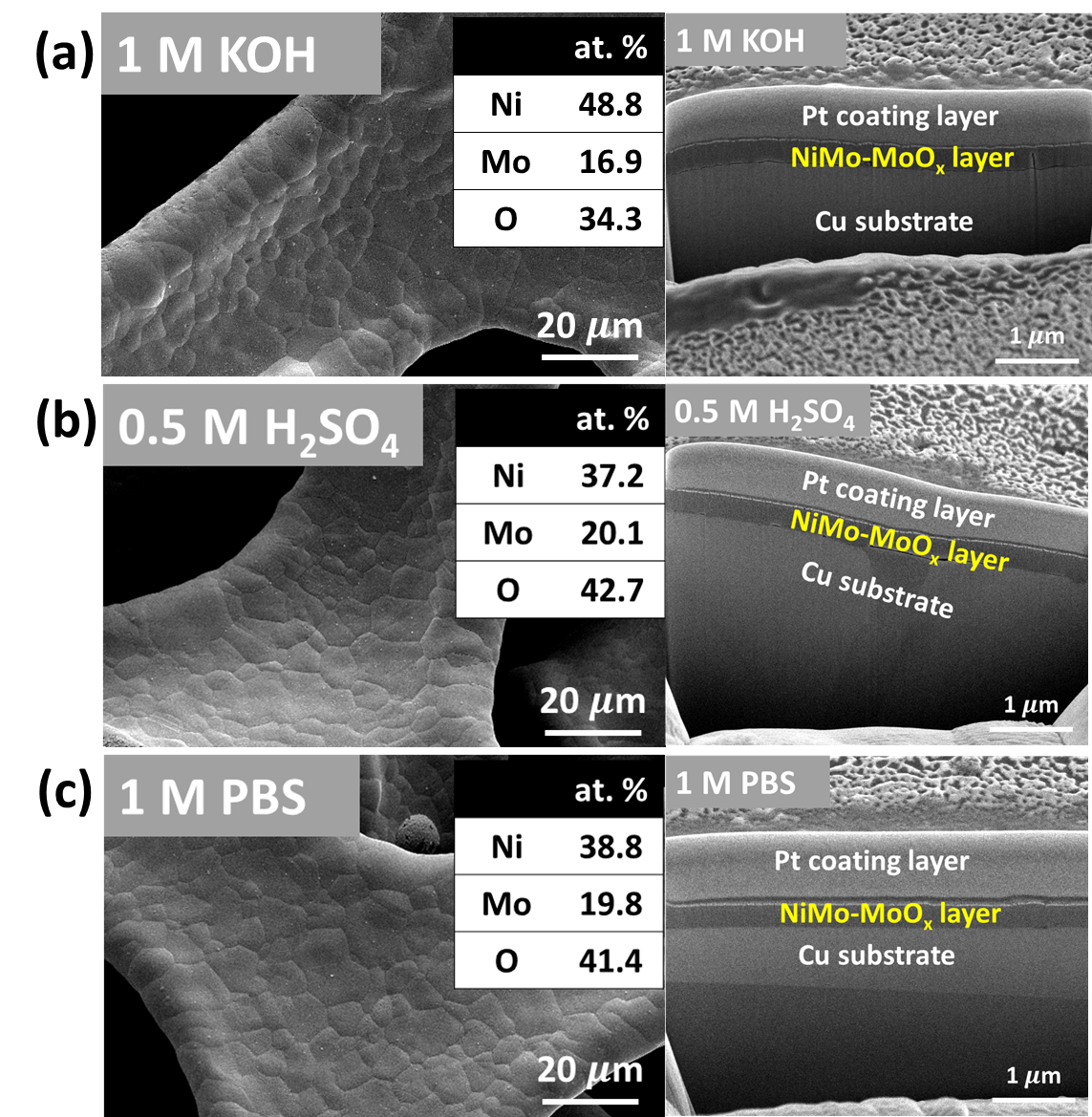
**

**Figure S14.** SEM images with EDS elemental composition and cross-sectional image of Ni-Mo(2.5) in (a) alkaline, (b) acidic, and (c) neutral electrolytes after the chronopotentiometry test for 20 h at -100 mA cm^-2^.


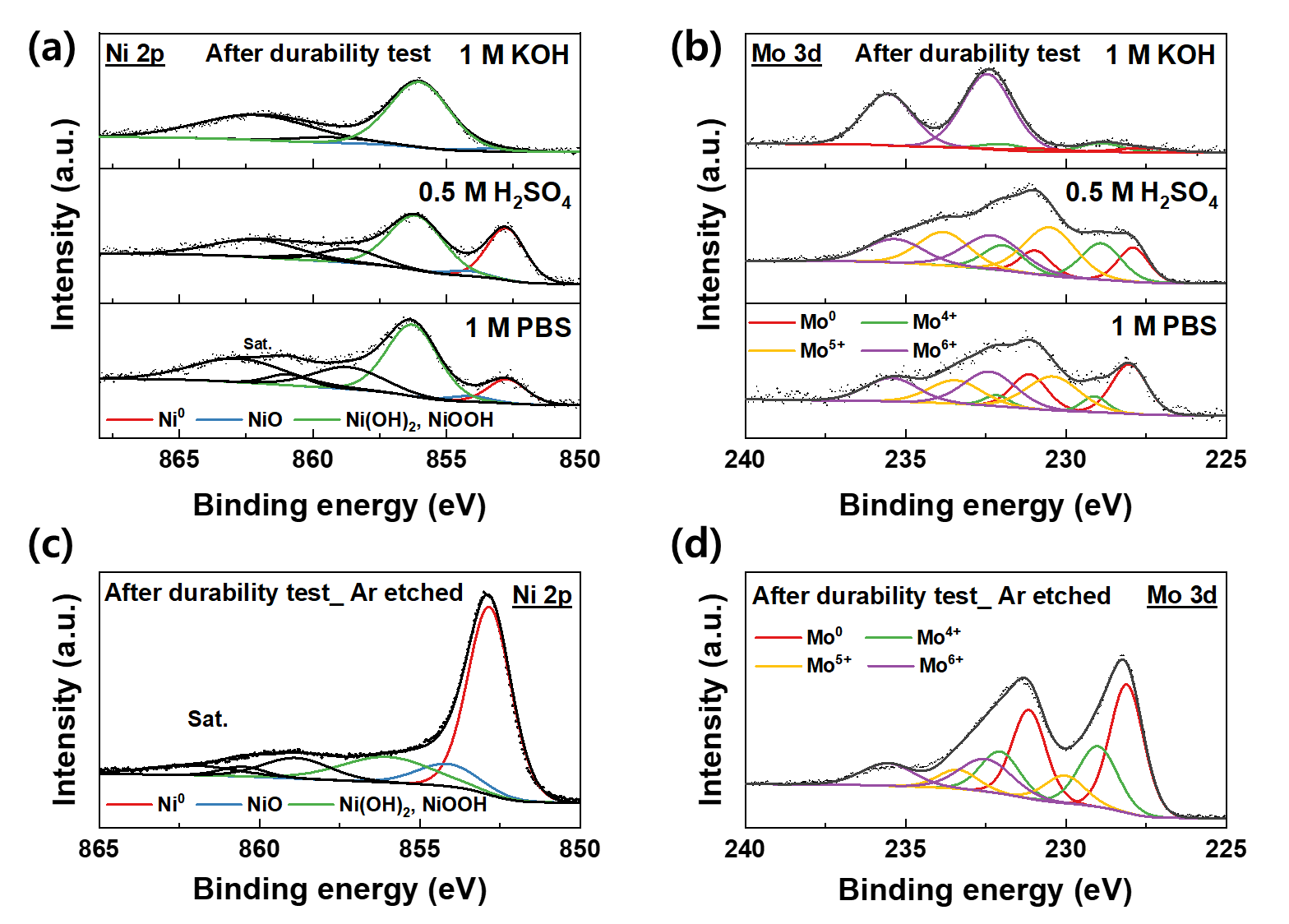


**Figure S15.** Post XPS analysis after the HER chronopotentiometry (@ -100 mA/cm^2^ for 20 h) for the Ni-Mo(2.5) catalysts: *ex-situ* XPS for (a) Ni 2p and (b) Mo 3d, surface-etched XPS for (c) Ni 2p, and (d) Mo 3d.


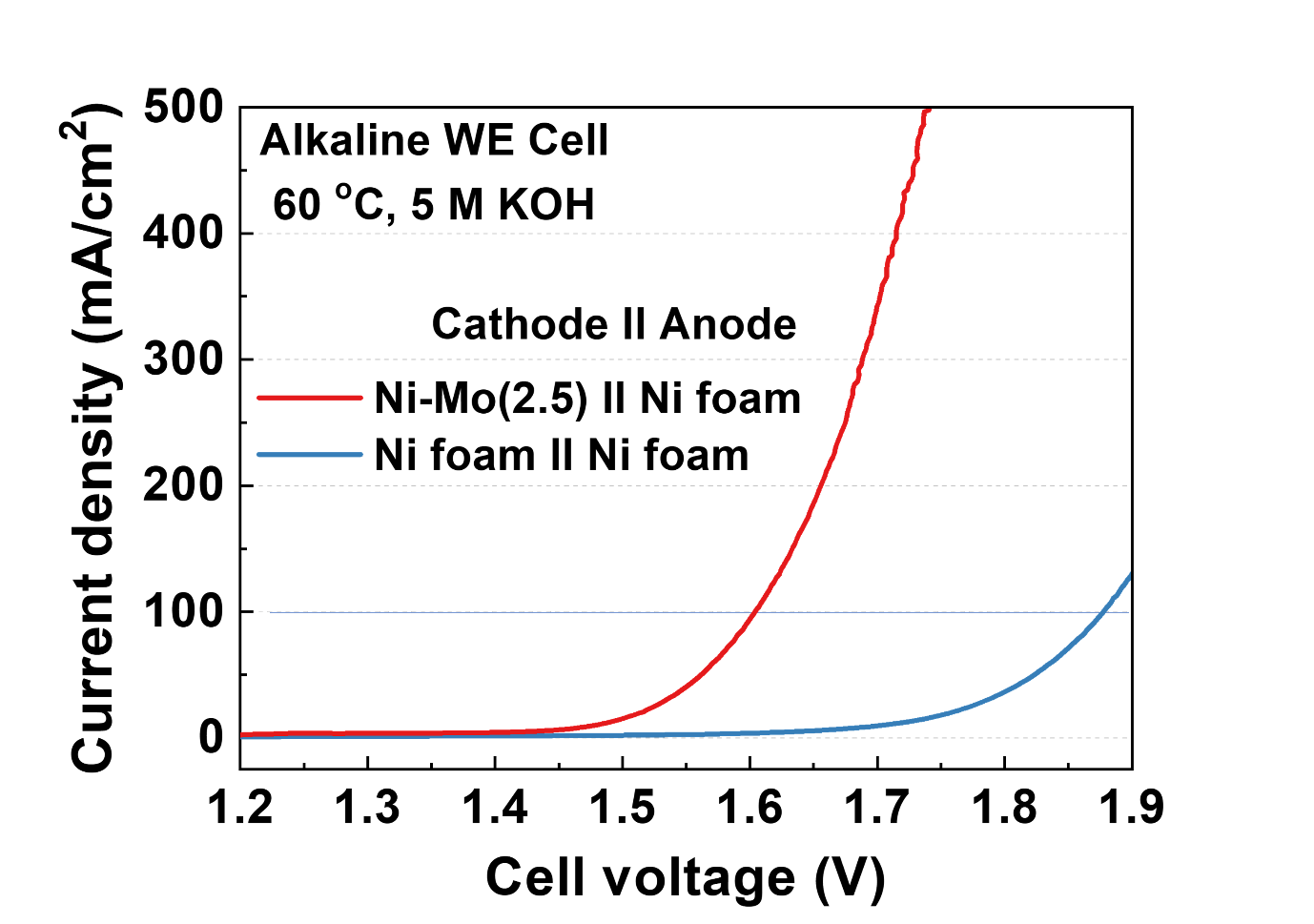


**Figure S16.** Alkaline water electrolysis cell performance using the Ni-Mo(2.5) as the cathode and Ni foam as the anode. The active area of the cathode was 1 cm^2^. The cell performance was measured from 1.2 to 2.55 V_RHE_ at a scan rate of 2 mV/s in 60 ^o^C 5 M KOH electrolyte.


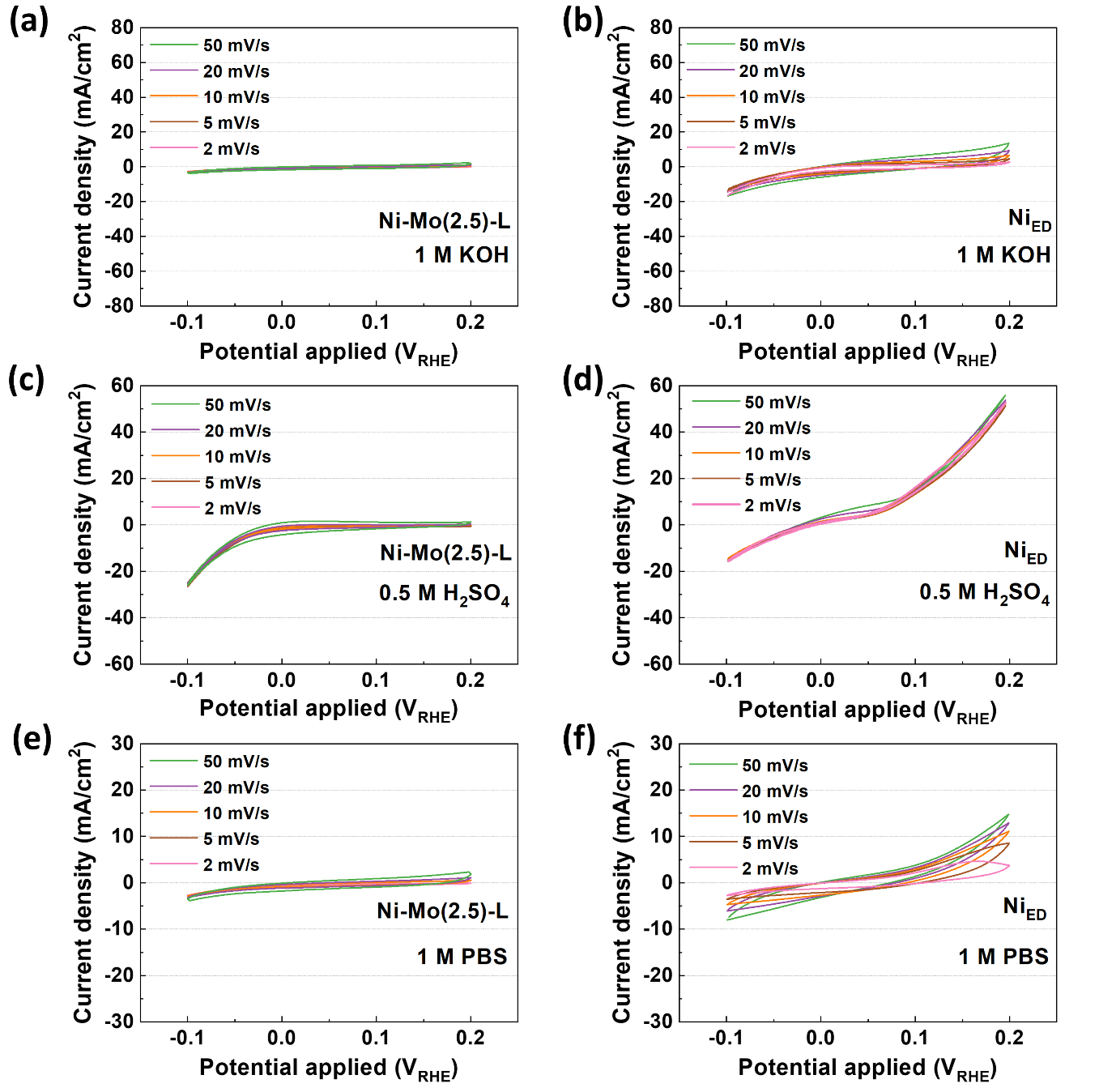


**Figure S17.** Cyclic voltammetry from -0.1 to 0.2 V_RHE_ for Ni-Mo(2.5)-L and Ni_ED_ in wide-pH electrolytes; (a, b) alkaline, (c, d) acidic, and (e, f) neutral, respectively.


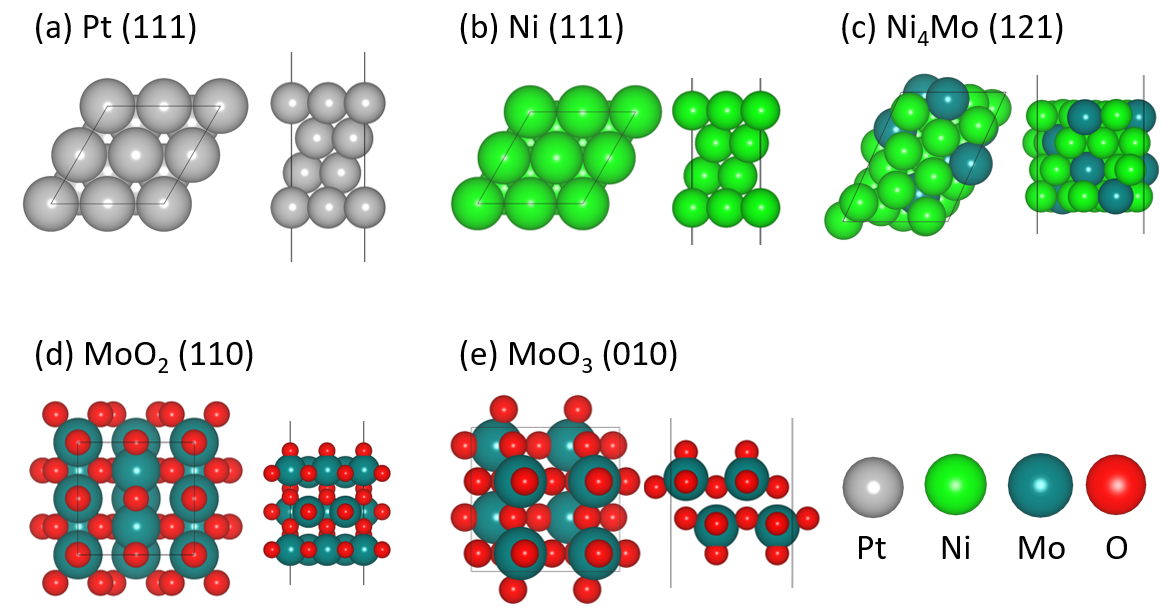


**Figure S18.** Modeling structure of (a) Pt (111), (b) Ni (111), (c) Ni_4_Mo (121), (d) MoO_2_ (110), and (e) MoO_3_ (010).


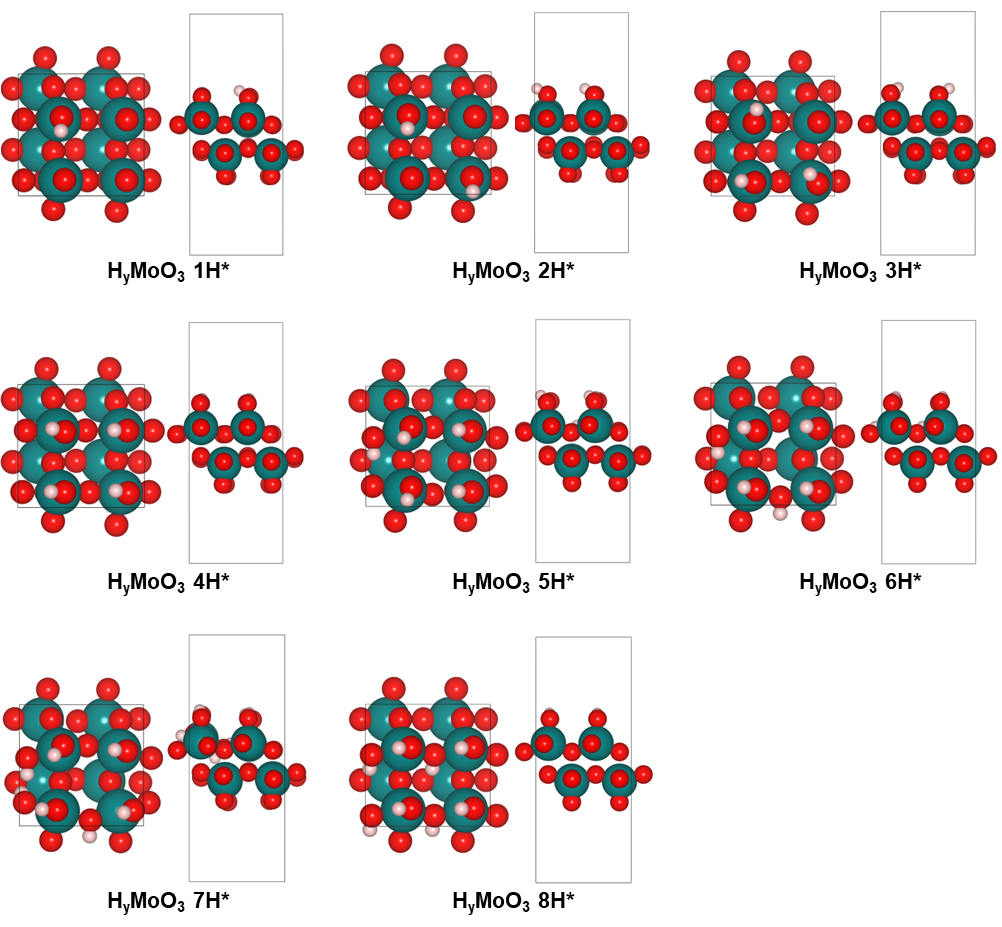


**Figure S19.** Theoretical structures of the hydrogen molybdenum bronze phase (H_y_MoO_3_-nH*, n = 1-8).

**
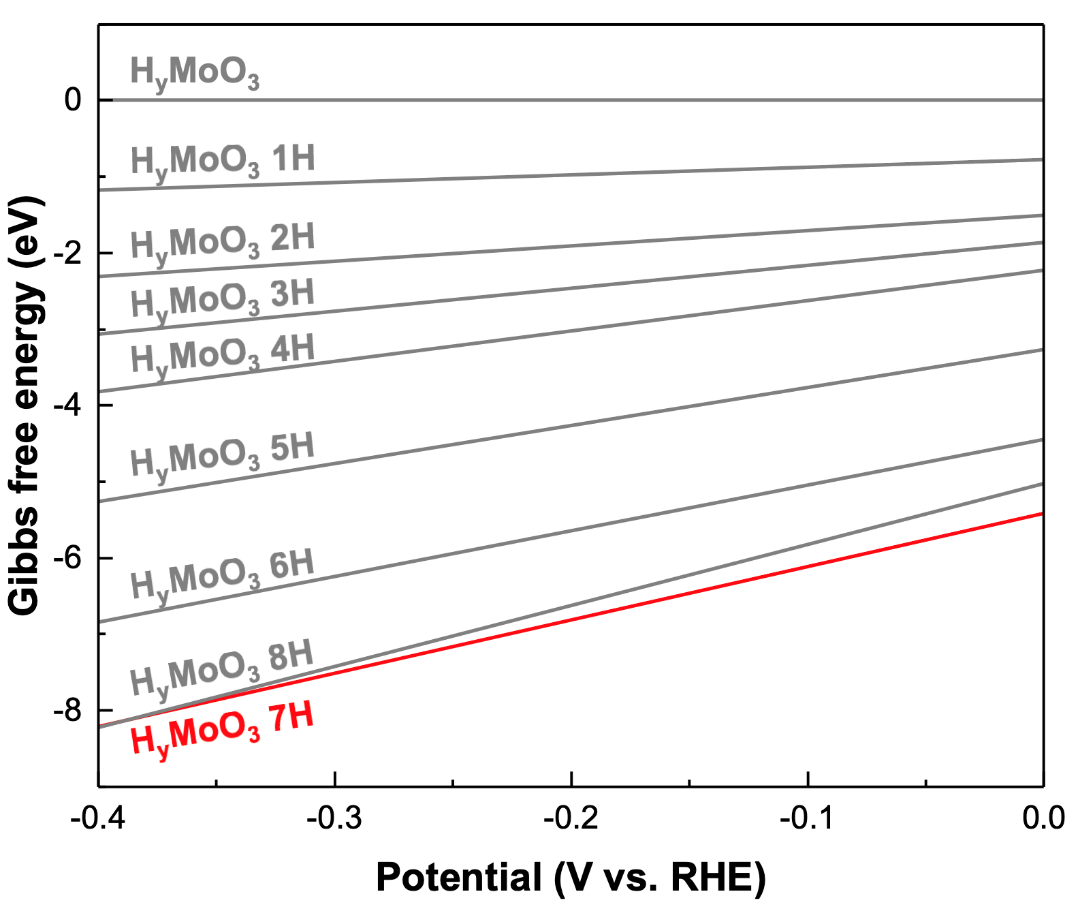
**

**Figure S20.** DFT calculated surface Pourbaix energy diagram for H coverage on the MoO_3_.


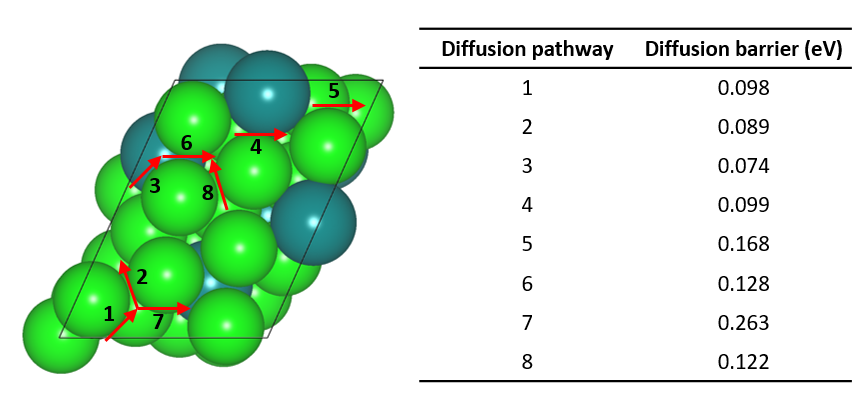


**Figure S21.** DFT-calculated diffusion barrier of hydrogen on the surface of Ni_4_Mo.

**Table S1.** The electrodeposition conditions for the preparation of the Ni-Mo binary electrodes.

| **Sample** | **NiCl_2_**  **(M)** | **Na_2_MoO_4_**  **(mM)** | **Na_3_C_6_H_5_O_7_**  **(M)** | **Current density**  **(A/cm^2^)** | **Time**  **(min)** |
| --- | --- | --- | --- | --- | --- |
| Ni_ED_ | 0.1 | 0 | 0.2 | 0.5 | 5 |
| Ni-Mo(1.25) |  | 1.25 |  |  |  |
| Ni-Mo(2.5) |  | 2.5 |  |  |  |
| Ni-Mo(5) |  | 5 |  |  |  |
| Ni-Mo(10) |  | 10 |  |  |  |
| Ni-Mo(20) |  | 20 |  |  |  |
| Ni-Mo(2.5)-L |  | 2.5 |  | 0.1 |  |
| Ni-Mo(2.5)-H |  | 2.5 |  | 1.0 |  |

**Table S2.** Electrochemical HER overpotentials of the Ni-Mo(2.5), Ni_ED_, and Pt/C electrodes at -10 mA·cm^-2^ (η_10_) and -100 mA·cm^-2^ (η_100_) in wide-pH electrolytes.

| **HER overpotential**  **(mV @ mA/cm^2^)** | **0.1 M KOH** | | **0.5 M H_2_SO_4_** | | **1 M PBS** | |
| --- | --- | --- | --- | --- | --- | --- |
|  | **η_10_** | **η_100_** | **η_10_** | **η_100_** | **η_10_** | **η_100_** |
| **Ni-Mo(2.5)** | 24 | 86 | 21 | 60 | 37 | 168 |
| **Ni_ED_** | 204 | 296 | 66 | 208 | 177 | 351 |
| **Pt/C** | 24 | 122 | 3 | 33 | 41 | 329 |

**Table S3.** The electrodeposition conditions for the preparation of the Ni-Mo binary electrodes.

| **Sample** | **Double-layer capacitance (C_dl_)**  (µF/cm^2^) |
| --- | --- |
| Ni_ED_ | 13.22 |
| Ni-Mo(1.25) | 14.49 |
| Ni-Mo(2.5) | 14.23 |
| Ni-Mo(5) | 14.27 |
| Ni-Mo(10) | 13.63 |
| Ni-Mo(20) | 14.67 |
| Ni-Mo(2.5)-L | 13.12 |
| Ni-Mo(2.5)-H | 14.70 |

**Table S4.** Electrochemical HER overpotentials of the Ni-Mo(2.5), Ni_ED_, and Pt/C electrodes after the chronopotentiometry test at -0.1 A·cm^-2^ for 20 h.

| **HER overpotential**  **(mV)** | **η_100_ in 0.1 M KOH** | | **η_100_ in 0.5 M H_2_SO_4_** | | **η_100_ in 1 M PBS** | |
| --- | --- | --- | --- | --- | --- | --- |
|  | **initial** | **after** | **initial** | **after** | **initial** | **after** |
| **Ni-Mo(2.5)** | 82 | 82 | 57 | 55 | 163 | 171 |
| **Ni_ED_** | 304 | 323 | 212 | 190 | 360 | 445 |
| **Pt/C** | 126 | 251 | 33 | 32 | 313 | 341 |

**Table S5.** Comparison of the HER activity with the Ni-Mo-based electrocatalysts in the literature.

| **Catalyst** | **Synthesis** | **HER overpotential at -10 mA/cm^2^ (ŋ_10_, mV)** | | | **Ref** |
| --- | --- | --- | --- | --- | --- |
|  |  | Alkaline | Acidic | Neutral |  |
| **Ni-Mo(2.5)** | **ED** | **24** | **21** | **37** | ***This work*** |
| Ni_2_Mo_3_N/NF | ED | 60 | N/A | N/A | 1 |
| NiFe/Fe-MoO_2_ | TD | 34 | N/A | N/A | 2 |
| Mo/Ni-P | ED | 79 | N/A | N/A | 3 |
| Ni/Mo-Ni | TD | 24 | N/A | N/A | 4 |
| Ni-Mo-O/PG | ED | 42 | 51 | N/A | 5 |
| O-NiMoP/NF | TD | 54 | N/A | N/A | 6 |
| Ni_2_Mo_6_S_6_O_2_/MoS_2_ | P | 90 | N/A | N/A | 7 |
| NiMo/Cu-NS/NF | ED | 89 | 43 | 86 | 8 |
| E-NiMo-7.5@0.02 | ED | 33 | 28 | 83 | 9 |
| Mo-Ni(OH)_2_ NSAs | TD | 22 | N/A | N/A | 10 |
| Mo-Ni-P | P | 50 | 58 | 77 | 11 |
| CoMoNiS-NF-31 | TD | 113 | 103 | 117 | 12 |
| NiMo-MoO_3-x­_-PNR | P | 24.5 | N/A | N/A | 13 |
| P-NiMoO_4_ | TD | 45 | N/A | N/A | 14 |
| NiS/NiMo | ED | 77 | N/A | N/A | 15 |

* Abbreviation: ED and TD indicate electro- and thermal-deposition, respectively. P indicates powder-based catalysts.

**Table S6.** DFT-calculated adsorption energy of hydrogen on the modeled systems (Pt, Ni, MoO_2_, MoO_3_, H_x_MoO_3__7H* (n=4, 6, 7, 8), and Ni_4_Mo)

| **Systems** | **Adsorption energy of H (eV)** |
| --- | --- |
| **Pt** | -0.08 |
| **Ni** | -0.23 |
| **Ni_4_Mo** | -0.30 |
| **MoO_2_** | 0.13 |
| **MoO_3_** | -0.53 |
| **H_y_MoO_3__7H*** | -0.16 |

**Table S7.** DFT-calculated adsorption/dissociation energy of H_2_O on the modeled systems (Pt, Ni, MoO_2_, MoO_3-x_ and Ni_4_Mo).

| **Reaction (eV)** | **Pt** | **Ni** | **MoO_2_** | **MoO_3-x_** | **Ni_4_Mo** |
| --- | --- | --- | --- | --- | --- |
| H_2_O 🡪 H_2_O* | 0.08 | 0.24 | -0.05 | -0.82 | -0.50 |
| H_2_O* 🡪 TS | 0.86 | 0.71 | 1.01 | 0.69 | 0.56 |
| TS 🡪 H*+OH* | -0.12 | -0.85 | 0.00 | -0.45 | -1.25 |

**REFERENCES**

1. Zhao, J. Y.; Lou, Z. X.; Xue, L. Y.; Ding, Y.; Li, X.; Wu, X.; Liu, Y.; Yuan, H. Y.; Wang, H. F.; Liu, P. F.; Dai, S.; Yang, H. G., Rational nitrogen alloying in nickel–molybdenum nitride can mediate efficient and durable alkaline hydrogen evolution. *Journal of Materials Chemistry A* **2023,** *11* (13), 7256-7263.

2. Shi, W.; Zhu, J.; Gong, L.; Feng, D.; Ma, Q.; Yu, J.; Tang, H.; Zhao, Y.; Mu, S., Fe-Incorporated Ni/MoO2 Hollow Heterostructure Nanorod Arrays for High-Efficiency Overall Water Splitting in Alkaline and Seawater Media. *Small* **2022,** *18* (52), 2205683.

3. Jiang, L.; Pan, Y.; Zhang, J.; Chen, X.; Ye, X.; Li, Z.; Li, C.; Sun, Q., Mo propellant boosting the activity of Ni-P for efficient urea-assisted water electrolysis of hydrogen evolution. *Journal of Colloid and Interface Science* **2022,** *622*, 192-201.

4. Li, H.; Cai, C.; Wang, Q.; Chen, S.; Fu, J.; Liu, B.; Hu, Q.; Hu, K.; Li, H.; Hu, J.; Liu, Q.; Chen, S.; Liu, M., High-performance alkaline water splitting by Ni nanoparticle-decorated Mo-Ni microrods: Enhanced ion adsorption by the local electric field. *Chemical Engineering Journal* **2022,** *435*, 134860.

5. Zhao, M.-J.; Li, E.-M.; Deng, N.; Hu, Y.; Li, C.-X.; Li, B.; Li, F.; Guo, Z.-G.; He, J.-B., Indirect electrodeposition of a NiMo@Ni(OH)2MoOx composite catalyst for superior hydrogen production in acidic and alkaline electrolytes. *Renewable Energy* **2022,** *191*, 370-379.

6. Jiang, H.; Sun, M.; Wu, S.; Huang, B.; Lee, C.-S.; Zhang, W., Oxygen-Incorporated NiMoP Nanotube Arrays as Efficient Bifunctional Electrocatalysts For Urea-Assisted Energy-Saving Hydrogen Production in Alkaline Electrolyte. *Advanced Functional Materials* **2021,** *31* (43), 2104951.

7. Cheng, H.; Diao, Y.; Liu, Q.; Wei, L.; Li, X.; Chen, J.; Wang, F., Di-nuclear metal synergistic catalysis: Ni2Mo6S6O2/MoS2 two-dimensional nanosheets for hydrogen evolution reaction. *Chemical Engineering Journal* **2022,** *428*, 131084.

8. Chang, Z.; Zhu, L.; Zhao, J.; Chen, P.; Chen, D.; Gao, H., NiMo/Cu-nanosheets/Ni-foam composite as a high performance electrocatalyst for hydrogen evolution over a wide pH range. *International Journal of Hydrogen Energy* **2021,** *46* (5), 3493-3503.

9. Park, J.; Kim, H.; Han, G. H.; Kim, J.; Yoo, S. J.; Kim, H.-J.; Ahn, S. H., Electrochemically fabricated MoO3–MoO2@NiMo heterostructure catalyst with Pt-like activity for the pH-universal hydrogen evolution reaction. *Journal of Materials Chemistry A* **2021,** *9* (6), 3677-3684.

10. Zhang, W.; Tang, Y.; Yu, L.; Yu, X.-Y., Activating the alkaline hydrogen evolution performance of Mo-incorporated Ni(OH)2 by plasma-induced heterostructure. *Applied Catalysis B: Environmental* **2020,** *260*, 118154.

11. Lai, C.; Liu, X.; Wang, Y.; Cao, C.; Yin, Y.; Yang, H.; Qi, X.; Zhong, S.; Hou, X.; Liang, T., Modulating ternary Mo–Ni–P by electronic reconfiguration and morphology engineering for boosting all-pH electrocatalytic overall water splitting. *Electrochimica Acta* **2020,** *330*, 135294.

12. Yang, Y.; Yao, H.; Yu, Z.; Islam, S. M.; He, H.; Yuan, M.; Yue, Y.; Xu, K.; Hao, W.; Sun, G.; Li, H.; Ma, S.; Zapol, P.; Kanatzidis, M. G., Hierarchical Nanoassembly of MoS2/Co9S8/Ni3S2/Ni as a Highly Efficient Electrocatalyst for Overall Water Splitting in a Wide pH Range. *Journal of the American Chemical Society* **2019,** *141* (26), 10417-10430.

13. Singu, B. S.; Chitumalla, R. K.; Mandal, D.; Kim, Y.; Kim, G. H.; Chung, H. T.; Jang, J.; Kim, H., Development of metal-organic framework-derived NiMo-MoO_3-x_ porous nanorod for efficient electrocatalytic hydrogen evolution reactions. *Applied Catalysis B: Environmental* **2023**, 328, 122421.

14. Zhang, K.; Su, Q.; Shi, W.; Lv, Y.; Zhu, R.; Wang, Z.; Zhao, W.; Zhang, M.; Ding, S.; Ma, S.; Du, G.; Xu, B., Copious Dislocations Defect in Amorphous/Crystalline/Amorphous Sandwiched Structure P-NiMoO_4_ Electrocatalyst toward Enhanced Hydrogen Evolution Reaction. *ACS Nano* **2024**, 18, 3791-3800.

15. Ren, T.; Huang, X.; Chen, J.; Wang, G.; Liu, Y.; Bao, F.; Guo, W., Surface-sulphated nickel-molybdenum alloy film as enhanced electrocatalysts for alkaline overall water splitting. *International Journal of Hydrogen Energy* **2024**, 57, 983-989.
